# An Insight into Recent Advances on Platelet Function in Health and Disease

**DOI:** 10.3390/ijms23116022

**Published:** 2022-05-27

**Authors:** Preeti Kumari Chaudhary, Sanggu Kim, Soochong Kim

**Affiliations:** Laboratory of Veterinary Pathology and Platelet Signaling, College of Veterinary Medicine, Chungbuk National University, Cheongju 28644, Korea; chaudharypreety11@gmail.com (P.K.C.); tkdrnfld@naver.com (S.K.)

**Keywords:** platelet, CVDs, inflammation, diabetes mellitus, cancer, wound healing, COVID-19

## Abstract

Platelets play a variety of roles in vascular biology and are best recognized as primary hemostasis and thrombosis mediators. Platelets have a large number of receptors and secretory molecules that are required for platelet functionality. Upon activation, platelets release multiple substances that have the ability to influence both physiological and pathophysiological processes including inflammation, tissue regeneration and repair, cancer progression, and spreading. The involvement of platelets in the progression and seriousness of a variety of disorders other than thrombosis is still being discovered, especially in the areas of inflammation and the immunological response. This review represents an integrated summary of recent advances on the function of platelets in pathophysiology that connects hemostasis, inflammation, and immunological response in health and disease and suggests that antiplatelet treatment might be used for more than only thrombosis.

## 1. Introduction

Platelets are anucleated, discoid cells with a diameter of 2 to 4 µm that develop from megakaryocytes in the bone marrow and are released into the circulation through pseudopodial projections called pro-platelets [1]. The physiological count of platelets in the circulation ranges from 150,000 to 450,000 platelets/µL of human blood, with a mean lifespan of 8 to 10 days. Platelets are metabolically active cells despite their absence of a nucleus because they include the endoplasmic reticulum, Golgi apparatus, and mitochondria, and they manufacture various proteins from mRNA. In particular, mitochondria have more functions in platelets compared to other cell organelles. Between 5 to 8 mitochondria are found in healthy platelets, the majority of which must stay intact for the platelet to operate properly [2]. Platelet mitochondria not only perform the fundamental mitochondrial function of ATP synthesis but also have been shown to contribute to platelet activation and apoptosis, making them far more significant than nucleated cell mitochondria [3]. The organelle zone of a resting platelet is made up of alpha granules, dense granules, lysosomal granules, and glycogen granules, and it includes about 1500 proteins involved in platelet activities, including 190 membrane proteins and 262 phosphoproteins crucial for platelet functional responses [4,5].

In vascular biology and homeostasis, platelets play a variety of roles. They are the earliest players in primary hemostasis and are critical in the development of thrombosis. In recent years, scientific research and technology have provided a new perspective on platelets and their functions due to their high granular content of growth factors (GFs), cytokines, and other biological modulators that can react to a wide range of signals and regulate a wide variety of biological processes including inflammation, angiogenesis, stem cell migration, and cell proliferation. These factors also exert a paracrine effect in different cell populations, including mesenchymal cells, osteoblasts, fibroblasts, and endothelial cells, and can lead to cellular proliferation, migration, and angiogenesis, all involved in various pathophysiology [6,7]. Acid proteases and glycohydrolases, among the digestive enzymes found in platelet lysosomal granules, have been demonstrated to play a role in the bactericidal activity, platelet desensitization via platelet aggregation clearance, and local destruction of the arterial wall’s connective tissue [8]. In addition, the existence of T-granule in platelet, a novel intracellular electronic dense granule in which TLR9 is expressed, has recently been revealed that is thought to respond to bacterial pathogen-associated molecular patterns (PAMPs), although its presence is uncertain due to lack of sufficient studies [9,10]. Thus, platelets, in addition to their essential involvement in hemostasis, play a pivotal role in other homeostatic processes. Platelet activation, adhesion, spreading, migration, aggregation, and stabilization forms a thrombus that becomes entangled in a developing fibrin network and entraps other blood cells. Recruitment of fibroblasts and immune cells through platelet-initiated angiogenic mechanisms eventually stimulates wound healing and tissue repair [11]. Using similar mechanisms, activated platelets also aggregate at the site of atherosclerotic plaque rupture, triggering thrombus formation and thereby inducing atherothrombotic disease [12]. Platelets are also part of innate immunity, which originates and accelerates a variety of inflammatory diseases. Their immunological activities are beneficial in certain situations, but they promote negative inflammatory effects in others. Platelets have a role in cancer development and spreading as a result of this property.

Despite breakthroughs in our knowledge of platelet function in diverse pathologies, many people continue to suffer from heart attacks, strokes, and other platelet-related disorders. Active participation in platelet biology research, from how they are generated to how they operate routinely and how they fail in disease, is critical, and new and present platelet-related sickness research should be encouraged.

In this review, we look in detail at platelets’ multifaceted involvement in both physiological and pathological situations, bridging hemostasis, inflammation, and immunological response in health and disease. We also discussed the various anti-platelet therapies currently used clinically in different disease conditions that would increase our understanding towards designing novel platelet-targeting therapeutic strategies in the future.

## 2. Principal Role of Platelet: Thrombosis and Hemostasis

Platelets, which have a substantial effect on hemostasis, act as the first line of defense in the body against bleeding after vascular injury. Under normal physiology, anti-platelets molecules, for example, nitric oxide (NO) and prostacyclin (PGI2), released by endothelium and glycocalyx surrounding endothelial cells prevent endothelial-platelet interaction [13,14]. After the damage to a blood vessel, a vascular spasm occurs and triggers vasoconstriction, which could eventually stop the blood flow. At this point, exposed collagen fibers and ECM release various cytokines, which recruit platelets and induce them to roll and adhere [15]. Von Willebrand factor (vWF), which binds to glycoprotein Ib-IX (GPIb-IX) in the platelet membrane, is responsible for platelet adhesion. This interaction allows platelet receptor GPVI to bind to collagen in the exposed extracellular matrix and delivers activation signals to platelets [16]. Following activation of platelets, they change their shape and release secretory granules including adenosine diphosphate (ADP), thrombin, serotonin, and epinephrine, and generating thromboxane A_2_ (TxA_2_), which recruit and activate additional platelets via their respective receptors [17]. Briefly, activated platelet membrane receptors including P2Y_12_, P2Y_1_, TP, and PARs stimulate their downstream signaling and trigger the integrin αIIbβ3 activation. Ligand binding to integrin αIIbβ3 induces platelet adhesion and aggregation, which activates inside-out signaling, which in turn activates outside-in signaling, triggering platelet spreading, further granule secretion, and the formation of the primary platelet plug. Then, fibrin, which is converted from fibrinogen via the intrinsic and extrinsic pathways, recruits additional platelets, builds fibrin mesh, and finally forms a secondary platelet clot [18]. Activated platelets constrict their cytoskeleton’s internal actin and myosin fibrils after the fibrin clot has formed, which leads the clot to compact and shrink. By converting plasminogen to plasmin, plasmin promotes fibrinolysis by cutting and destroying the fibrin network. Blood flows in injured or obstructed blood vessels are restored by the clot resolution process [18].

## 3. Contribution of Platelets beyond Thrombosis and Hemostasis

**Figure 1:**
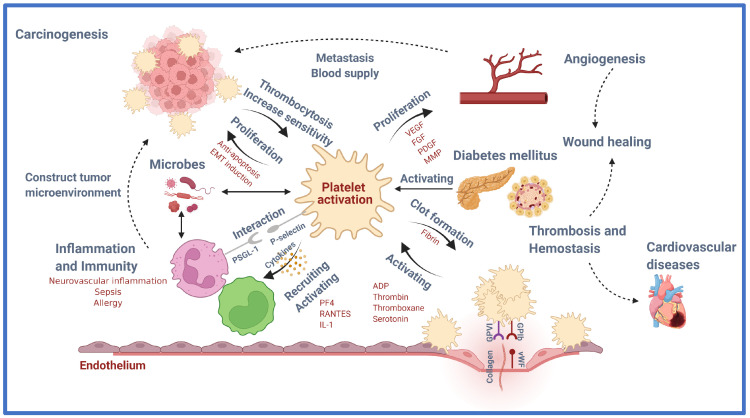
Role of platelet in health and disease. Platelets are versatile cells engaged in numerous pathophysiological processes including inflammation and immunity, angiogenesis, regeneration, and carcinogenesis, in addition to their crucial role in thrombosis and hemostasis via various molecular and cellular events. This figure introduces the emerging role of platelets in the immune system, vascular biology, tumorigenesis, and beyond. VEGF, vascular endothelial growth factor; FGF, fibroblast growth factor; PDGF, platelet-derived growth factor; MMP, matrix metalloproteinase; EMT, epithelial-mesenchymal transition; PF4, platelet factor 4; RANTES, regulated upon activation, normal T cell expressed and presumably secreted; IL-1, interleukin 1; vWF, von Willebrand factor; ADP, Adenosine diphosphate. Created with BioRender.com. Available online: https://app.biorender.com/illustrations/626bc5c233d53778dbd97902 (accessed on 19 May 2022).

Platelets were once thought to have exclusively hemostatic activity, but recently, scientific studies and technology have discovered a link between platelet function in hemostasis/thrombosis and diseases including cancer, inflammation, and neurological problems (Figure 1).

Platelets are currently emerging as critical regulators of these numerous physiological and pathological processes, in addition to their well-known role in regulating bleeding and thrombosis. Platelets’ amazing capacity to control such a wide variety of physiological and pathological processes is largely due to their granules and microparticles’ (MP) ability to store and release a wide range of biologically active chemicals (Table 1). Platelets also exhibit biochemical and functional variability, with various platelet subpopulations, including the procoagulant platelet type [19]. The various functions of platelets are possibly due to this apparent customization of function. Platelets are no longer considered “band-aids” for the circulatory system, which is understandable. This review highlights some of the most recent advances and discoveries in the pathophysiological roles of platelets beyond thrombosis and hemostasis as explained below:

### 3.1. Contribution of Platelet to Cardiovascular Diseases (CVDs)

CVD is the major cause of death and is rising at an alarming rate of 17.3 million individuals a year globally. The primary pathophysiological mechanism for the development of CVD is atherosclerosis. Platelets are now widely acknowledged to have a key role in the early stages of endothelial dysfunction in the atherosclerotic process that leads to plaque rupture at the final stages, subsequently causing CVD. Research studies have suggested platelet activity varies among different individuals, which may justify the heterogeneity of CVD [69]. In conjunction, thrombosis, inflammation, and atherogenesis are associated with promoting and altering inflammation and the immune response in the body. A wide variety of molecules such as chemokines, pro-inflammatory molecules, and other biological response modulators released from platelet granules, after binding to the injured vascular endothelium and activation, enhance the association between platelets, endothelial cells, and leukocytes [70]. The localized inflammatory response that facilitates the atherosclerotic process is formed by these interactions. Table 2 lists the functions and pathways of platelet molecules involved in CVD.

Briefly explaining, in atherosclerosis, platelets have an immunomodulatory function and communicate with different receptor-ligand inflammatory cells. Monocytes interact with activated platelets and transform myeloid-related protein (MRP-14) into these cells and macrophages [123]. Platelets play a critical role in CVD lipid metabolism, and lipids can activate inflammatory factors in platelets. Platelets contain peptide hormone receptors that can be triggered resulting in thrombosis [69,81]. CXCL7, a chemokine that is expressed highly in platelet expression, is shown to have a function in CVD [124]. Similarly, the CXCL12/CXCR4-CXCR7 axis modulates the platelet lipidome (such as triacylglycerols and ceramides), which might be involved in pathophysiological function in CVD [125,126]. Oxidized-LDL and platelet surface binding are said to elicit activation, morphological alterations, and platelet aggregation that leads to thrombosis formation [127]. Elevated levels of sphingomyelin regulate the catabolism of sphingolipids in CVD and induce platelet hyper-reactivity. Increased ceramide is linked with platelet activation and sphingosine-1-phosphate changes blood sphingolipid levels [76]. Inflammation, endothelial dysfunction, monocyte, and macrophage differentiation, plaque formation, and ischemic conditions are characterized by oxidized phospholipids that stimulate pro-inflammatory genes and thrombosis via CD36 scavenger receptor and platelet-activating factor receptor (PAFR), tissue factor (TF), and TFPI [76,128]. LDL adhesion stimulates platelet receptors such as CD36, resulting in ROS formation and platelet activation in CVD patients [75,128]. LDL can stimulate platelets through the CXCR4-CXCR7 axis, which activates apoptosis and inflammatory responses in monocytes with CXCL12, resulting in atherogenesis [129]. Platelet-secreted chemokines (such as CXCL12) can contribute to inflammation and initiate autocrine and paracrine responses and thrombosis. LDL on platelets is associated with platelet numbers, showing that platelet activation, prothrombinase complex aggregation, thrombin formation, and thrombosis can be influenced by plasma lipids [74]. Thus, aiming these receptors on platelets can introduce a better plan for managing CVD.

Similarly, peptide hormone proteins such as leptin are located on platelets and are capable of activating platelets and increasing thrombosis. Experiments have reported leptin activates long-form leptin receptor (LEPRL), Janus kinase 2 (JAK2), phosphatidylinositol 3-kinase (PI3K), protein kinase B (PKB), insulin receptor substrate-1 (IRS-1), and PDE3A signaling cascade. When this pathway is activated, platelet PDE3A rises, and the inhibitory function of cAMP in platelets declines [81,85]. Insulin resistance relates to platelet activation, and hypoadiponectinemia and platelet-leukocyte aggregations (PLAs) are seen in CVD patients to indicate atherosclerosis. An increase in platelet phospholipids and ROS production has been linked to CVD pathological process.

In inflammatory disease, the immune system has a remarkable part in the development of atherosclerosis. The platelets further express the receptors of P-selectin, integrins such as GP IIb/IIIa, and the expression of Toll-like receptors (TLRs) for immunological functions. Leukocytes, endothelial cells, and SMCs with various receptors and mediator secretions such as ADP, TxA_2_, and cytokines such as IL-1β can also be activated by platelets. In addition, MPs are formed by activated platelets in the circulation. In patients with the ACS, reports have revealed that certain secretory proteins increase MPs during platelet activation [130]. In inflammation and CVD, miRNAs play a major role. The miRNAs are found in regulatory platelets and MPs have many miRNAs that allow platelets to interact with vascular cells, contributing to inflammation and vascular homeostasis. Through lipid metabolism regulation, inflammation, cell proliferation, angiogenesis, and platelet activation, miRNAs regulate the pathogenesis of ACS and CVD.

Likewise, WDR1 binding to the collagen matrix increases platelet adhesion and activation, which exerts more prothrombotic activity, and it has been hypothesized that the risk of CVD is enhanced [80,131]. Preeclampsia is a significant source of maternal and perinatal morbidity and mortality that affects 3–5% of pregnant women worldwide and is correlated with platelet activation and CVD development in adults [132]. As per the data, the equilibrium between PGI2 (a vasodilator and platelet inhibitor) and TxA_2_ (a platelet activator and vasoconstrictor) in preeclampsia is changed [133]. Thus, there is a greater danger of developing hypertension, atherosclerosis, and CVD in patients with prior preeclampsia in later life and an increased possibility of cardiovascular death [134].

In conclusion, platelet activation and thrombosis are complex processes, and there are various molecular pathways associated. The chemicals that activate platelets have been proposed as indicators for anticipating the treatment outcomes of CVD and could be used to prevent thrombosis and atherosclerosis. The variability in platelet activation would also have an effect on the development of atherosclerosis, thereby influencing CVD. Inhibiting these molecules, on the other hand, can govern platelet interactions, thrombosis, and CVD by reducing platelet activation and aggregation. For example, the P2Y_12_ receptor blocker (clopidogrel) has been shown to improve the inhibition of coronary thrombosis and decrease CVD. Low dose clopidogrel or acetylsalicylic acid (ASA), and FXa inhibitors have been effective for CVD patients. In addition, thrombin and FXa inhibitor dual antiplatelet treatment tend to show favorable impacts. In ACS and coronary stenting patients, dual antiplatelet treatment with ASA and clopidogrel revealed better outcomes for one year [135]. In contrast, ASA-treated dual ticagrelor and vorapaxar-treated PAR-1 block the activity of platelet thrombin, do not coagulate, increase bleeding relative to ASA alone, and do not impact CVD [136,137]. Therefore, in CVD patients, adequate anticoagulant doses have to be calibrated against the benefits and risks. Besides that, simultaneous antiplatelet and anticoagulant therapy in these patients relies on the safety of the anti-coagulant medication to control extreme bleeding. It is feasible to consider desensitizing the platelet to regulate CVD. Since platelets are engaged in all stages of the atheromatic process, their significance as a therapeutic target is important from initiation to clinical complications. Furthermore, the recognition of platelet-derived mediators as clinical diagnostic biomarkers will lead to a greater perception of atherosclerosis and CVD pathophysiology and will allow us to better treat this condition with fewer unnecessary side effects.

### 3.2. Platelet in Diabetes Mellitus (DM)

Diabetes is a complex illness that is linked to micro- and macrovascular problems including systemic inflammation, endothelial dysfunction, cardiovascular risk as well as a significant risk of atherothrombotic events. Platelets play a key role in the development of inflammation and cardiovascular risk, and thus, play a critical part in the pathogenesis of DM. DM patients’ platelets do have dysregulation of numerous signaling cascades that lead to platelet activation, which is a prior occurrence in the disease’s natural history (neatly reviewed by Francesca Santilli et al., 2019 [138]). The abnormalities in platelet function found in DM are assumed to be caused by the harmful metabolic state such as acute hyperglycemia, glycemic fluctuation, and insulin resistance that leads to and escorts diabetes. These metabolic deviations may influence platelet transcriptome and/or posttranscriptional modulation through intermediate mediators such as oxidative stress with isoprostane formation, inflammatory molecule production, endothelial dysfunction with circulating endothelial cells and MPs release, and cross-talk between cells with miRNA exchange through circulating MPs [139]. Platelet hyperreactivity is a consequence of these impacts, as seen by increased platelet TF expression and TF-positive PLAs; increased expression of adhesion molecules such as P-selectin; increased expression and hyperactivity of P2Y_12_ receptor; increased expression of platelet GPIb and GPIIb/IIIA; dysregulation of thrombin and fibrinogen level; and increased arachidonic acid metabolism and accelerated TXA_2_ production. Platelets from diabetes individuals had lower amounts of cAMP, resulting in increased P2Y_12_ signaling and greater baseline intracellular calcium levels, and additional accelerated calcium mobilization from intracellular reserves in response to thrombin agonism [140]. Platelets from diabetic patients, unlike those from healthy people, showed short-term activation of the calcium-sensitive protein kinase C (PKC) β isoenzyme by acute hyperglycemia in vitro. In diabetics, the number of platelet MPs has also been reported to be higher [141]. Insulin can directly modulate platelet function via a functional insulin receptor present on human platelets [142]. Dysregulated and circulating inflammatory molecules such as tumor necrosis factor α (TNF-α), IL-1, and IL-6 have been shown to activate the release and expression of procoagulant molecules, such as vWF, PAI-1, and TF, and inhibit the expression of anticoagulant molecules, such as thrombomodulin, by endothelial cells, creating an environment suitable for hyper-activation of the platelet [143]. These hyperactivated platelets, in turn, play a critical part in the progression and spreading of long-term inflammation in diabetes, are progressively being identified as the cells responsible for the increased risk of atherothrombosis in the diabetic setting, and add to diabetes vascular complexity. All of these complications are significantly accelerated at the late stage of diabetes. In the late or advanced stages of DM, the metabolism is affected, thereby causing various complications affecting almost every part of the body and is associated with macrovascular complications such as atherosclerosis and stroke. Additionally, chronic hyperglycemia has been shown to affect platelet function by impairing calcium homeostasis, thereby altering platelet activation and aggregation, including platelet conformation [144] and release of mediators and eventually leading to atherosclerosis, heart attack, stroke, and other complications related to poor circulation. Thus, platelets seem to be both targets and effectors in the pathogenesis of DM, transporting and transducing metabolic abnormalities into vascular damage.

Antiplatelet drugs such as aspirin and clopidogrel have been used in DM patients but were shown to have a poorer response to these drugs due to a hyper-activated platelet signaling system [145]. Interestingly, metformin has been shown to reduce ADP-induced platelet aggregation in insulin-dependent diabetes by reducing activated platelet-induced mitochondrial hyperpolarization, ROS overload, and mitochondrial DNA release [146].

Furthermore, despite a wealth of data on platelet sensitivity to a range of aggregating agents in vitro in type 2 DM (T2DM), it is questionable if these abnormalities are caused by circulating substances that alter platelet function, as insulin immunocomplexes have shown [147]. In reality, abnormal platelet function in DM appears to be linked to a number of variables, including metabolic changes, oxidative stress, hyperlipidemia, inflammatory state, and endothelial dysfunction and is thought to be influenced by a number of pathways induced by metabolic and cellular abnormalities. An in vitro investigation of washed platelet activity in diabetes patients would be fantastic for elucidating the role of platelets in disease development. Additionally, population-based studies of platelet aggregation in diabetes are limited as samples are frequently selected on the basis of comorbidities such as CAD or disease severity and should be designed. Further, attempts to reduce the thrombotic load in diabetes should focus on particular disease-based processes in the future. In this context, high-throughput approaches are critical because they provide a distinctive chance to comprehend the molecular networks affected by T2DM, such as platelet transcriptome and proteome composition and/or post-transcriptional modulation. Likewise, diabetes staging plays an important role in the prevention, diagnosis, and treatment of diabetes, according to experts in the field of diabetes research. Therefore, understanding the effect of platelets on various stages of diabetes can improve its long-term management. Platelet function tests can also be utilized to assess the effectiveness of treatment regimens in DM patients as clinicians have a variety of therapeutic choices when platelet dysfunction is discovered. In conclusion, platelet investigation in DM is an essential area to investigate in order to find new treatment targets, and platelets might be exploited as cellular activity monitors themselves.

### 3.3. Platelet in Wound Healing

Wound healing might be one of the world’s most serious issues. Healing or replacing damaged tissues is a complex process involving biological, physical, and chemical obstacles. In wound healing, a variety of cell types, GFs, cytokines, and active metabolites are all coordinated in real-time. Wound healing necessitates hemostatic system components such as coagulation factors and platelets. As a matter of fact, platelets are a natural supply of GFs, and they also produce a variety of other chemicals, such as fibronectin, vitronectin, and sphingosine 1-phosphate, that aid in tissue maintenance, regeneration, and repair. Hemostasis, inflammation, proliferation, and remodeling/maturation are some of the sequential processes that have been established to delineate such a complicated process of wound healing. Platelets are involved in all of these stages, from the beginning, when they are the most numerous cell type, until the end [148]. Platelets construct a fibrin clot that halts bleeding, act as a temporary scaffold for inflammatory cells, and comprise a reserve of cytokines, chemokines, and GFs which influence the beginning stages of repair, such as the enrollment of neutrophils as the first line of defense against pathogens. Indeed, after 12–24 h of the following damage, neutrophils make up half of the cells in the wound, but macrophages take over after 3–5 days [149]. Neutrophils and platelets collaborate to resolve inflammation by producing pro-resolving mediators and polarizing macrophages toward a repair phenotype [150]. Platelets also produce several growth and angiogenic compounds, which contribute to the proliferative phase. Neovascularization is a critical procedure for healing tissue to fulfill its high metabolic needs, and it is carefully modulated by the secretion of pro- and anti-angiogenic molecules in a balanced manner. Platelets activate the assignment of CD34+ bone marrow-derived endothelial progenitors through the release of SDF-1, in addition to sprouting angiogenesis induced by the release of VEGF, HGF, and FGF [151]. Many cellular protagonists in healing, including keratinocytes in wounds and bone cells in fractures, might benefit from the arsenal of GFs housed in platelet granules. Furthermore, PDGF and platelet-released TGFs operate on fibroblasts to replace the first temporary fibrin scaffold with granulation tissue rich in immature collagens, fibronectin, and proteoglycans [152]. Platelets also help to reconstruct the extracellular matrix by secreting MMPs and releasing hydrolases from their lysosomes. GPIbα binds the integrin macrophage antigen 1 (Mac-1), which is expressed on leukocytes, in addition to vWF. Notably, heterotypic cell-cell contacts between leukocytes and platelets promote pro-inflammatory and pro-thrombotic processes [153]. For fibroblasts, platelet-derived PDGF is mitogenic and motogenic, and it promotes neutrophil recruitment. TGF-β is produced in large numbers from platelets shortly after injury, and this first burst of active TGF-β acts as a chemoattractant for neutrophils, macrophages, and fibroblasts [154]. EGF from platelets promotes keratinocyte migration, fibroblast function, and granulation tissue development [155]. The proof that microgravity impedes receptor binding and signal transduction exacerbates the reduced GF release. Healing outcomes are determined by fibrin structure.

A number of studies demonstrate the advancement of tissue repair and wound healing upon platelet-rich plasma (PRP) administration. These findings, together with the ease with which PRP may be obtained, suggest that PRP might provide a new opportunity in the area of tissue repair. The reason for employing PRP is based on findings that platelets produce GFs when they degranulate, speeding up the assignment of cells involved in the healing process. For its efficacy in soft tissue healing, PRP treatment has shown great outcomes in oral and maxillofacial surgical operations [156]. Our growing understanding of platelet function in wound healing and tissue regeneration has led to the success of autologous PRP-derived products, which are now widely used in a variety of clinical settings, ranging from skin wounds and diabetic ulcers to tendons and ligament regeneration, from eye lesions to bone loss [157]. For example, PRP has been suggested as a local source of cytokines and GFs in patients with symptomatic osteoarthritis in various hyaluronan-controlled and placebo-controlled clinical trials [158]. PRP has anti-inflammatory properties, enhances cartilage height, and lowers cartilage matrix loss by inhibiting chondrocyte death [159]. The release of GFs, cytokines and extracellular matrix modulators promotes (i) revascularization of injured tissues through the appointment of endothelial cell migration, proliferation, differentiation, and stabilization in new blood vessels; (ii) recovery of ruptured connective tissue through fibroblast migration, proliferation, and activation; and (iii) proliferation and differentiation of mesenchymal stem cells. For these reasons, PRP derivatives are utilized in regenerative medicine to treat a variety of ailments. However, because of their main autologous origin, there is little risk of disease transmission or immunogenic responses. In the recent decade, platelet-enriched materials have grown more important in regard to wound healing and tissue regeneration, and they have been a rising focus of experimental and clinical research.

Despite the wide range of uses, the efficacy of PRP-based regeneration therapies is being questioned due to a lack of large-scale controlled clinical trials and a lack of consensus on PRP preparation methodologies. The cellular and molecular mechanisms of PRP as well as the potentially harmful effects and effectiveness of these therapies remain poorly understood. Although platelets secrete antiangiogenic factors, their levels are insignificant in comparison to the high levels of antiangiogenic molecules present in plasma, which contains both matrix-derived and nonmatrix-derived inhibitors (e.g., angiostatin, soluble version of VEGF receptor (VEGFR) -1, pigment epithelium-derived factor (PEDF), and prolactin), all of which can compete with and conversely interfere with a variety of GFs receptors. In regard to regenerative therapies using PRP, more study is required to determine its probable contribution.

### 3.4. Platelet in Inflammation and Immunity

#### 3.4.1. General Role of Platelet in Inflammation and Immunity

Inflammation is a response of the innate immune system to pathogenic stimuli like pathogens, or damage-related molecular patterns (DAMPs) and is thought to be aided by platelets.

When activated, platelets release a variety of cytokines like IL-1α, IL-1β, CD40L, TNF-α, CXCL1, CXCL5, and CXCL12 to induce inflammation including leukocyte migration, phagocytosis, and ROS generation. Activated platelets also cause the release of several chemokines like PF4 (CXCL4), CXCL7, IL-8 (CXCL8), CCL3, CCL5, CCL7, CCL17, which induce inflammation by recruitment of leukocytes through their chemotactic activity [160,161]. The main platelet-derived mediators of inflammation are listed in Table 3.

The formation of ROS and oxidized LDL by activated platelets cause the expression of VWF on the endothelial cell, as well as endothelial dysfunction [181]. Endothelial cells produce PGI2, NO, and ecto-ADPase (CD-39) under normal physiological conditions, which prevents platelet adhesion to intact endothelium [182,183]. Excessive ROS and LDL cause endothelial dysfunction, which converts the internal vascular surface from a non-adhesive barrier to one that recruits leukocytes, amplifies platelet aggregation and accelerates the inflammatory process of leukocyte adhesion [184].

Platelets and leukocytes do not interact with each other under normal physiological conditions. Their interlinkage becomes practical in a prothrombotic or proinflammatory state with an increasing number of blood PLAs found in disorders such as DM, stroke, and others [185,186]. This interaction begins with the binding of P-selectin on activated platelets to P-selectin glycoprotein ligand 1 (PSGL-1) on leukocytes, which initiates a signaling pathway inside leukocytes that results in the activation of integrins on the leukocyte membrane, particularly Mac-1 and LFA-1 on the leukocyte membrane [163,187,188]. Mac1 can directly bind platelet receptors GPIbα or αIIbβ3 through fibrinogen bound to the integrin on platelets [189,190]. Outside-in signaling regulates multiple leukocyte functions such as transmigration and the production of ROS when the integrins are fully activated [191]. The activation of Mac-1 by platelets might result in the sequestration and activation of coagulation Factor X leading to the production of thrombin generation [192]. Platelet-monocyte aggregation also increases the production of monocyte cytokines including CCL2, IL-6, and TNF-α, as well as the release of platelet agonists such as platelet-activating factor (PAF), collagen, thrombin, TxA_2_, leading to the augmentation of inflammation and platelet activation [162]. All of these phenomena result in platelet-leukocyte interaction, which can contribute to diseases associated with various inflammation-related diseases such as rheumatoid arthritis [193], multiple sclerosis (MS), Alzheimer’s disease (AD) [194], and allergic response [195] as well as thromboinflammatory diseases such as atherosclerosis [193], ischemia [196], sepsis [197,198], and stroke [199], and lastly, thrombocytopenia is identified.

Platelets affect not only innate immunity but also adaptive immunity. Platelet-derived molecules (PF4, serotonin, TGF-β) have been shown to enhance differentiation and antigen presentation in DCs. In addition, platelets can induce the recruitment of lymphocytes to the site of inflammation [200,201]. Moreover, platelets express CD40L, C-type lectin-like receptor II-type (CLEC-2), and P-selectin, which can increase interaction and activation of antigen-presenting cells (B cells, DC) as well as T cell activation. Major histocompatibility complex (MHC) class I is present in platelet α-granules which present antigen to CD8+ T cells. Proteasomes in platelets, such as immunoproteasome subunit β5 [202], play a role in adaptive immunity by hydrolyzing proteins into smaller peptides and loading them into the MHC class I [203,204].

So far, several treatments for inflammation that target platelets have been attempted. Rapamycin, an inhibitor of mammalian target of rapamycin (mTOR) pathways, reduces platelet inflammatory reactions in the prophylaxis of organ rejection transplant patients [205]. Furthermore, rapamycin has recently been discovered to have anticoagulant properties by protecting mitochondrial integrity independently of mTOR, suggesting that it may be more effective for the treatment of platelet-induced inflammation [206]. Inhibition of P38 mitogen-activated protein kinase (MAPK) by RNA interference may lower IL-1β synthesis and lead to reduced inflammation [194]. The canakinumab anti-inflammatory thrombosis outcome study (CANTOS) trial revealed that the therapeutic monoclonal IL-1β antibody canakinumab had a favorable impact on myocardial infarction [207]. Tofacitinib, the first rheumatologic JAK inhibitor, has suppressive effects on PDGFs in vascular smooth muscle as well as an anti-platelet effect [208,209]. Statins and aspirin have been demonstrated to decrease the quantities of MPs generated by platelets and megakaryocytes, which are responsible for transmitting inflammatory signals [210,211]. In addition, abciximab, an αIIbβ3 inhibitor that inhibits the synthesis of Bcl-3 and IL-1β, thereby reducing excessive platelet accumulation, is predicted to be used [212,213].

Multiple studies have also reported that platelet derivatives have inflammatory side effects. Since platelets have a significant impact on adaptive immunity as well as innate immunity, a pathological understanding of platelets in immunopathological conditions is essential, which could lead to the target of new immunological treatments. Additionally, although targeting platelets for the treatment of inflammation may be effective, it may also inhibit platelet activation, resulting in coagulation failure, and too much inhibition may inversely lead to immune suppression, causing another complication. To be an effective treatment, research on the safe zone for candidates for platelet-targeted inflammatory treatment and cocktail therapy must be done in advance.

#### 3.4.2. Platelet in Sepsis

Sepsis is an uncontrolled multi-pathogenic infection-related systemic response that includes immune and coagulation system dysregulation, thrombosis, disruption of endothelial barrier function, increased vascular permeability, microvascular sequestration, tissue destruction, and more [53]. It can result in severe sepsis causing multiple-organ failures and cognitive impairment. In the pathophysiology of sepsis, platelets are both cellular effectors and cellular targets. Platelets have a critical part in the development of multiple-organ failure, regardless of the initial events in sepsis, due to their hemostatic and thrombotic potential, culminating in thrombotic microangiopathy and disseminated intravascular coagulation (DIC) [197,214,214]. Even though it is yet uncertain if the decline in platelet count is the cause or a result of sepsis severity, persistent thrombocytopenia is considered an independent risk factor for death in sepsis. The involvement of platelets in the onset and progression of sepsis has been studied in a variety of experimental in vivo models, most notably mice, which mimic various elements of human sepsis.

The host’s pro-coagulant and pro-thrombotic condition is exacerbated by sepsis. Endotoxins, both local and circulating, cause endothelial cells and monocytes to produce tissue factor (TF), which promotes intravascular fibrin deposition and vascular blockage. Furthermore, microbial infections cause neutrophils to produce neutrophil extracellular traps (NETs), that offer a negatively charged surface for coagulation factor activation and assemblage [215]. The coagulation cascade’s activation creates a positive feedback loop that causes platelet activation via thrombin production, boosting micro thrombosis in response to inflammation and infection. Immunothrombosis is a portion of the antimicrobial host response that seeks to entrap invading pathogens and stop them from spreading, albeit it is likely pathogen- and organ-dependent [216,217]. Furthermore, the active endothelium boosted platelet recruitment and adhesion, resulting in the production of microthrombi throughout the body [218]. Platelets can be trapped in the capillary-rich microvasculature of the spleen and liver during sepsis. The bulk of platelets, on the other hand, concentrate in the microvasculature of the lungs [219,220]. Excessive immune responses in sepsis are frequently accompanied by increased and dysregulated coagulation and thrombosis, which manifests as DIC. Microthrombi easily grow inside tiny and medium arteries during DIC, causing tissue oxygenation to be disrupted, multi-organ failure, and finally circulatory collapse. Through their part in inflammation and thrombosis, activated platelets aid in the development and progression of sepsis. Although many different processes contribute to the severity and persistence of thrombocytopenia, sepsis is typically followed by a reduction in platelet count, indicating their sequestration and consumption in microthrombi [218]. A dysregulated host response is linked to severe thrombocytopenia, which results in an increase in cytokine levels and endothelial dysfunction [221,222]. As a result of the utilization of coagulation factors and platelets, sepsis is linked to increased systemic thrombosis and coagulation, as well as an increased risk of bleeding.

Platelets express a variety of receptors including pathogen identification receptors, immune cell activation receptors, and platelet activation receptors that are critical in the onset and course of sepsis. While many receptors are located on all platelets, some are exclusively found on certain subpopulations of platelets (e.g., TLRs [223]). Additionally, circulating platelets vary in terms of age, maturity, and density. It is largely unclear if receptor content tends to vary during platelet formation as a result of platelet maturation, differences in thrombopoiesis, and receptor distribution, or whether a portion of megakaryocytes produces immune-regulatory receptor-expressing platelets while others generate platelets that only serve thrombotic functions [224]. Clinical research and animal model studies showing the therapeutic effect of antiplatelet medications in sepsis give evidence for platelets’ importance. Septic individuals have changes in their circulating platelets [225,226]. In some investigations, expression of CD62P was higher in septic platelets, but not in others [227,228]. Membrane expression of thrombospondin and CD63, raised soluble CD40L level, and a rise in β-thromboglobulin (β-TG) and the β-TG-to-PF4 ratio are further platelet activation indicators seen in sepsis [226,228,229]. The production of VEGF from septic patient’s platelets stimulated by agonist was shown to be increased [227]. Furthermore, an activating receptor expressed on myeloid cells TLT-1 is present in the plasma of patients with sepsis at levels that correlate with DIC [230,231]. TLT-1 is released upon platelet activation and is found in the plasma of septic patients at concentrations that correlate with DIC. Again, soluble TLT-1 promotes adhesion of platelet to the endothelium [232] and has a role in the control of inflammation in sepsis by reducing activation of leukocytes and influencing platelet-neutrophil crosstalk [233]. Septic patients’ platelets are shown to be hyper-adhesive to cultivated endothelium [234]. Sepsis has been linked to changes in the aggregation of platelets and an increase in PLA levels, both of which may contribute to inflammation and vascular damage [225,235]. During sepsis, several platelet-activating mechanisms are likely to work together. Some, but not all, bacteria or bacterial products, as well as NETs, activate platelets [236]. This might help in the battle against infection (pathogen capture within the thrombus, pathogen killing). Unlimited thrombus development in reaction to bacteria or NETs, on the other hand, might be harmful in sepsis.

In virally infected animals, TLR7 activation causes platelet degranulation and PLA formation, as well as altering survival [237]. In platelets, TLR9 controls foreign DNA sequestration and CD62P surface expression [238]. TLR4 is implicated in the fast production of TNF [223], the creation of NETs [239], and thrombocytopenia [240]. TLR4 activation stimulates the production of neuraminidase during gram-negative (but not gram-positive) infection, boosting alkaline phosphatase clearance and increasing lipopolysaccharide phosphorylation and toxicity [241]. In septic mice, injection of neuraminidase after Streptococcus pneumoniae infection enhances survival and reduces the incidence of fibrin clots, as well as liver and spleen damage. In addition, neuraminidase causes mild thrombocytopenia suggesting that moderate thrombocytopenia may be advantageous in sepsis [242]. Many bacteria, including Streptococcus sanguis, have a serine-rich protein A (SrpA) that is acknowledged by GPIb and permits platelet-bacteria interaction in a sialic acid-dependent way [243]. Spa binds to vWF, which facilitates indirect platelet contact via GPIb in Staphylococcus aureus [244]. Several bacterial proteins, notably SdrG from Staphylococcus epidermis, can bind to the arginine-glycine aspartic acid (RGD) sequence on GPIIb/IIIa, triggering platelet aggregation [245]. Borrelia burgdorferi attaches to human platelets through the GPIIb/IIIa system [246]. On Staphylococcus aureus, clumping factors (Clf) bind fibrinogen, causing platelet aggregation [247]. FcRIIA promotes immune complex-induced platelet activation or opsonized bacteria death in infectious situations [248,249].

In septic patients, platelet activation is increased, which is amplified in septic shock and is linked to enhanced CD62P, CD63, and CD31 surface expression, increased fibrinogen binding, and soluble GPVI [250], especially in patients with DIC [251]. Furthermore, individuals with multiple-organ failures have higher thrombospondin expression on circulating platelets [234]. Platelets from sick patients aggregate spontaneously, but their ex vivo responsiveness to platelet agonists is significantly diminished [252]. In septic patients, activation of platelets is also linked to increment in platelet-neutrophil and platelet-monocyte aggregates, further amplifying the inflammatory response. These findings show that platelets in septic patients circulate activated, boosting their thrombotic propensity. Due to the intricacy of platelet activation in sepsis, several receptors are likely to be involved, making it more probable that combination treatment will be necessary to prevent the activation of platelets in sepsis. However, the increased risk of bleeding in the patients complicates platelet targeting in septic patients.

Antiplatelet medications such as aspirin (cyclooxygenase (COX) -1 inhibitor), platelet P2Y_12_ receptor antagonists such as clopidogrel, or GPIIb/IIIa antagonists have been proven in several observational and retrospective clinical trials to lower mortality and morbidity in critically sick patients [253,254,255]. P2Y_12_ inhibitors suppress pro-inflammatory and pro-thrombotic pathways in human experimental endotoxemia [256]. Furthermore, in septic patients, low-dose aspirin is linked to a lower risk of death during hospitalization as they spend less time in the hospital and require less intensive care [257]. In a large cohort study of septic patients, taking aspirin for 24 h at the time of systemic inflammatory response syndrome (SIRS) detection was linked to a higher rate of survival [258].

In conclusion, because platelets play a crucial part in thrombosis and inflammation, inhibiting platelet activity in sepsis is an appealing target. However, caution must be used when attempting to target platelets in infection. To demonstrate a definite positive benefit of anti-platelet medications in sepsis, large randomized controlled clinical studies using anti-platelet treatment are required.

#### 3.4.3. Role of Platelets in Neurovascular Inflammation

The central nervous system (CNS) is immune-favored, with the blood-brain barrier (BBB) separating it from the rest of the body. BBB can be disturbed in pathological situations. This allows blood cells to enter brain tissue, facilitating both innate and adaptive immune responses in the CNS [259,260]. Platelets, endothelium, and leukocyte interactions begin and maintain inflammation of larger arteries as well as the microcirculation of neural tissues. Cell-cell interactions and paracrine mediators are involved in this reaction. Platelets are seen in the inflamed CNS microvasculature of mice and can activate brain endothelial cells. Platelets have a role in brain disorders linked with pathogen-induced and sterile inflammation as inflammatory cells [261,262]. Platelet activation in neuroinflammation might be due to the direct detection of damaged tissue structures. For example, systemic treatment of sialated glycosphingolipids (gangliosides), components of astroglial and neuronal lipid rafts of the BBB, caused significant platelet activation and degranulation in rats. Platelets have been shown to detect the cerebral gangliosides GT1b and GQ1b specifically, with P-selectin playing a key role [263].

Migraine, the third most common condition in the world, is caused by sterile inflammation and pain pathway hypersensitization [264]. Platelet activation and aggregation are known to occur spontaneously in migraine patients [265], and fibrinogen and serotonin receptors in platelets are changed in migraine patients [266]. The accumulation of PLA in the blood of migraine sufferers may be a relationship between severe migraines and stroke [267]. Antiplatelet treatments may be useful in reducing the intensity of migraine [268]. MS and stroke are two neurological disorders that are linked to vascular inflammation. According to growing research, platelets appear to have a significant role in these scenarios. When MS patients were compared to healthy people, platelet activation indicators were shown to be increased in MS patients. Platelets may be important for illness development and severity via influencing leukocyte recruitment as one possible underlying mechanism since platelets were observed to have a direct role in leukocyte rolling and adherence to the endothelial cells of inflamed postcapillary venules via the GPIb-Mac-1 interaction [269]. The severity of the condition was significantly reduced by blocking platelet function. Clinical symptoms in animal models of experimentally induced autoimmune encephalomyelitis (EAE) were shown to be linked to PAF levels [270]. This conclusion was in line with the findings of rising PAF levels in the cerebrospinal fluid (CSF) of patients with relapse MS [271]. Platelets have also been found in CNS lesions in both MS patients and EAE animals [269]. Following EAE development, mice produced more mRNA for platelet surface antigens GPIb and GPIIb [269]. Therapeutic inhibition of platelet GPIbα may reduce the harmful consequences of excessive inflammation while also reducing the risk of thrombocytopenia-related bleeding problems in the brain [272]. Whenever the BBB is breached after a brain injury, platelet-neuron synapse-like structures form, producing serotonin and leading to increased neuronal plasticity and postsynaptic density protein (PSD)-95 development in dendritic spines [273].

Strokes result from localized cerebral ischemia induced by arterial embolism in around 80% of cases. Cerebral embolism starts in the heart in around one-third of ischemic stroke patients, especially in those with atrial fibrillation. As a result, the molecular pathways for coronary artery and pathogenic thrombus generation in myocardial infarction and stroke are thought to be partly intersecting. Inflammatory cytokines such as C-reactive protein, IL-6, and coagulation factors have exhibited considerable interaction in cerebral venous thrombosis (CVT), suggesting that inflammation has an essential role in thromboembolic illness. Inflammatory cells in unsteady plaques might express procoagulant chemicals (particularly tissue factor), which may stimulate coagulation by binding factor VIIa, which creates thrombin (factor IIa), which activates platelets and leads to platelet-fibrin thrombosis. In diseases of the CNS, inflammation, and thrombosis have a complex reciprocal connection.

Platelet function is disrupted in AD, and platelet activation status is thought to be a biomarker for disease progression [274,275]. In contrast, a recent study of platelet migration into the brain parenchyma in an AD mouse model found just a few platelets moving into the brain parenchyma [276]. In addition, just a few platelets were directly found in the brain parenchyma of AD patients’ histological specimens [276]. Platelets, on the other hand, were shown to be related to Alzheimer’s plaques or arteries near Alzheimer’s plaques in people [276].

Several pathways for the production of thrombus have previously been well discovered in the ischemic brain [199]. Blocking the GPIbα and GPVI receptors on platelets has been shown to reduce infarct sizes dramatically without changing platelet count, and improve neurological function [277]. When VWF knockout mice were given transient middle cerebral artery occlusion (tMCAO), the volume of their strokes was decreased by 60% with respect to wild-type mice, and this was also translated into a better functional outcome [278].

Platelet-derived IL1 appears to be particularly important in neurovascular inflammation of the brain. Primary mouse brain endothelia exposed to platelets separated from wild-type mice show dramatically enhanced expression of the surface antigens VCAM-1 and ICAM-1, as well as release meaningful levels of CXCL-1, but platelets isolated from IL1-/- animals had no such impact. Furthermore, IL1 raised the expression of VCAM-1 and ICAM-1 on brain endothelia, which was followed by enhanced neutrophil transendothelial migration [279].

To summarize, thromboinflammation is firmly linked to neurovascular disorders and has a substantial part in the progression of the illness. Other inflammatory cells, such as microglia and platelets, and their sequelae thrombosis and inflammation, play a major role in neurovascular illness in addition to conventional immune cells implicated in disease pathology and tissue destruction. Although the importance of platelet activation and the coagulation pathway in the development of CNS illness is known, a possible crosslink in a platelet-specific setting or functional relevance has yet to be addressed. Understanding how platelets contribute to tissue death, as well as regeneration of injured neural tissue, might lead to new and critically needed therapeutic techniques that go beyond the present and tragically restricted therapy options. More in-depth experimental and clinical investigations will be required if a function for platelets in vascular inflammation can be generalized to other neurological illnesses. Following these creative and original approaches and bringing together experts from many disciplines of study may be beneficial. Furthermore, focused antithrombotic treatment techniques to minimize the severity and brain damage in MS, stroke, or other neurovascular illnesses without raising the risk of bleeding might be a useful supplementary therapy.

#### 3.4.4. Platelet in Allergic Inflammation

Allergic illnesses are a group of ailments induced by immunological reactions to antigens in the environment. For many years, researchers have looked at the relationship between platelet activation, platelet abnormalities, and allergies such as asthma, allergic rhinitis, and eczema [280,281]. In atopic dermatitis and psoriasis patients, higher platelet activation blood markers such as β-TG, PF4, P-selectin, and platelet-derived MPs in plasma have been shown [282]. The activation of platelets and eosinophils in the airways of asthma patients has been found to have a substantial relationship [283]. Platelets are shown to be required for leukocyte recruitment in allergic inflammation of human and murine lungs after exposure to allergens [284,285]. Furthermore, the presence of extravascular platelets in the lungs of asthma patients and animal models of allergic lung inflammation shows that platelets may directly impact allergic inflammation by altering lung function or influencing airway wall remodeling processes. Platelets produce a number of physiologically active mediators when IgE receptors are activated, including RANTES, a powerful eosinophil chemoattractant [286]. IgE present in platelet granules might increase allergic reactions [240]. In 1972, Benveniste et al. discovered that leucocyte-dependent histamine release from platelets included activation of IgE, which led to the discovery of PAF, a lipid mediator [287]. Moreover, the major source of serotonin produced during an allergic inflammatory response in mice is platelets, not mast cells. PF-4 levels are shown to be considerably greater in individuals with severe asthma than in non-severe asthma, suggesting that the degree of platelet activation may rise as disease severity increases [288]. The localized migration of platelets to the lungs is a potential consequence of platelet activation that leads to peripheral thrombocytopaenia.

Gallagher and colleagues found that when platelets extracted from allergic patients during allergy season were tested for their reactivity ex vivo, they commonly displayed impaired secondary wave platelet aggregation [289]. Others have corroborated platelets’ apparent lack of reactivity to aggregatory stimuli, as well as the fact that mediators stored within platelets were reduced in asthma patients [290]. Platelets were thought to be acting in an ‘exhausted’ state, in which they had been engaged by inflammatory stimuli in vivo and released mediators, but were then resistant to further activation ex vivo [289,290]. Since then, several studies of altered platelet behavior in allergic illness patients have surfaced, varying from altered arachidonic acid metabolism to increased turnover of intracellular signaling cascades. Patients with allergic asthma exhibit a modest hemostatic deficiency and delayed thrombin production [291]. Despite considerable platelet activation in allergic illness patients, it is worth noting that these individuals are defined as having a minor hemostatic defect rather than a higher risk of thrombosis [292]. Furthermore, platelet lifetime has been found to reduce from 8.9 days to 4.7 days in stable atopic asthmatic patients, indicating that platelet consumption is ongoing as a result of chronic activation [293]. Treatment with corticosteroids was also shown to enhance platelet turnover, correcting the observed platelet lifetime decrease in atopic asthmatic patients [294]. Other studies have found no changes in platelet lifespan between healthy people and asthma patients, or pulmonary platelet sequestration after induction of allergen, implying that the processes governing platelet production, transit, and lifespan in the context of inflammatory disease are complicated [295,296]. Changes in mean platelet volume (MPV) and mass, for example, have been suggested as possible indications of changes in platelet production or consumption, which might correspond with differences in platelet function or activation [291].

Despite their widespread use, anti-platelet medicines particularly targeting platelet activation during hemostasis and thrombosis have no documented effect on asthma patients. Conversely, medications such as disodium cromoglycate (DSCG), which have long been recognized to be therapeutically useful in allergic illnesses, have been demonstrated to suppress certain platelet’s inflammatory effects both in vitro and ex vivo without affecting platelet aggregation [297]. This discrepancy in platelet function shows that platelet aggregation is controlled by different platelet activation pathways, as opposed to platelet activation produced by inflammatory mediators, which results in enhanced motility, platelet-leucocyte conjugates, and inflammatory mediator release. While research into these inflammatory-dependent activation cascades is still in its early stages, increasing understanding of these pathways might lead to the development of innovative anti-inflammatory and anti-asthmatic medications. Furthermore, platelet activities involving aggregation and those involving immunological processes, including communication and interactions with leukocytes, platelet chemotaxis, and direct antimicrobial effects, have unique physiological signals and mechanisms [298]. This has led to the concept of a platelet activation dichotomy—coagulation vs. platelet participation in a variety of physiologic immune responses, and also inflammatory illnesses such as allergy diseases and asthma [299].

### 3.5. Platelet in Malignancy

Platelets are energetic participants in every stage of carcinogeneses such as tumor development, tumor cell extravasation, and metastasis, according to experimental findings [300,301].

Platelets have the ability to transport a variety of proangiogenic factors to the tumor, such as VEGF, PDGF, FGF, and MMPs, and increase tumor cell production of proangiogenic factors. VEGF, which is stored in α-granules and activates VEGFR, promotes NO production and PGI2 via activating PI3K/AKT signaling and MAPKs, respectively. Vascular permeability, vasorelaxation, and endothelial survival are all improved by PGI2 and NO [302]. VEGF also enhances endothelial cell sprouting and tube formation by inducing mitogenesis and migration in these cells [303]. The recruitment of SMCs and pericytes, as well as the stability of new capillaries, need the presence of PDGF [304]. FGF stimulates endothelial cell proliferation and physical organization into tube-like structures, as well as blood vessel-associated cell growth, differentiation, and survival [305]. MMPs regulate angiogenesis by degrading matrix and activating or secreting GFs (FGF, VEGF) to provide favorable conditions for endothelial and tumor cell migration [306,307]. Several MMPs are also responsible for atypical vascular remodeling and invasion at secondary metastatic locations [308]. Platelets help to promote neovascularization, which ensures a sufficient supply of blood for providing nutrients, eliminating waste, and oxygenating the tumor [301].

Furthermore, pro-inflammatory chemokines including CXCL, MCP1, CCL3, CCL5, CCL7, CCL17, and PAF, recruit a range of immune cells to create tumor cell-platelet emboli. These chemokines successfully construct tumor microenvironments, resulting in enhanced vasculature, subversion of immunity, and tumor facilitation [309].

By surrounding the circulating tumor, platelets assist the tumor to evade the immune system and TNF-α-mediated cytotoxicity. Platelet layers protect circulating cancer cells from high shear forces at the vascular wall and aid thrombus formation, which helps to maintain tumor blood vessel integrity [310]. Platelet-tumor cell aggregation transfers normal MHC-1 molecules onto the surface of tumor cells, which prevents natural killer cells from recognizing cancer cells [300,301]. Platelets’ capacity to shelter circulating tumor cells from immune surveillance is believed to have a part in the metastatic process.

In metastatic cancer patients, increased platelet numbers and high rates of platelet activation are frequent [311]. During cancer progression, tumor-related humoral factors and cytokines affected platelet formation through direct paracrine influence on megakaryocytes and their capacities to educate and activate platelets. Granulocyte colony-stimulating factor (G-CSF) and granulocyte-macrophage colony-stimulating factors (GM-CSF) are the most well-known agents that stimulate megakaryopoiesis and thrombopoiesis [312]. Moreover, platelets boost the capacity of platelets to release pro-angiogenic proteins, such as VEGF, following activation of platelets by primary tumor education in the case of breast cancer [313]. Many solid tumors, such as pancreas, renal, and colorectal cancer [314,315,316], can display thrombocytosis, or raised platelet count, which is associated with a worsened prognosis because platelets promote tumor development [317].

In both hematological and solid tumor cancers, platelets have an anti-apoptotic function [300,301]. Platelets suppress apoptosis via enhancing Ras homolog family member A (RhoA)-myosin phosphatase target subunit 1 (MYPT1)- protein phosphatase 1 (PP1)-mediated yes-associated protein (YAP1) dephosphorylation and MAPK signaling in cancer cells [318,319]. Platelets also triggered EMT in cancer cells by activating the TGF/suppressor of mothers against decapentaplegic (Smad) dependent pathway via platelet-produced TGF and the nuclear factor-kB (NF-kB) pathway via direct platelet-tumor cell interaction [320]. These EMTs help tumor cells spread and metastasize by participating in angiogenesis sprouting, reinforcing the stromal fibroblastic milieu, and remodeling the vasculature [321]. Even platelets contribute to chemotherapy resistance through tumor proliferation, anti-apoptosis, and EMT induction [315]. TGF-derived from activated platelets enhances MAPK and PI3K/AKT signaling in pancreatic cancer cells, reducing cisplatin sensitivity [322]. In the membrane of dense granules, platelets produce significantly high levels of multidrug resistance-associated protein 4, which has been linked to a poor chemotherapy response [323]. Platelets scavenge anti-angiogenic drugs such as sunitinib and bevacizumab, which keep angiogenesis going [324].

There is less information about the effectiveness of platelets on benign tumors than on malignant tumors. A study reported that in ovarian tumors, the levels of IL-6 and platelets are greater in malignant tumors compared to benign tumors [325]. In contrast, alpha granule secretion, such as beta-thromboglobulin and PF-4, and platelet aggregation revealed no difference between ovarian cancer and benign ovarian tumors [326,327]. Furthermore, in the case of PRP supplementation in benign breast cancer, malignancy progression through metastasis of sarcoma phenotype emerges through a change in the microenvironment [328]. These data suggest that platelets may have a role in transforming benign tumors into malignant ones. However, a detailed future study with various cancer types in the presence and absence of platelet or platelet derivative may add knowledge to the present literature and elucidate the exact mechanism involved.

Platelet-targeted tumor therapy can be successful since platelets contribute to tumor growth. Aspirin, a typical COX-1 inhibitor, inhibits the conversion of arachidonic acid into prostaglandins, reducing platelet activation [329], and abrogating tumorigenic and metastatic effects [330,331]. Clopidogrel [332,333,334] and ticagrelor [335], among other antiplatelet medicines, greatly reduced cancer metastasis and improved survival rate. Antiplatelet therapy combined with chemotherapeutic therapy may improve therapeutic efficacy by lowering resistance, but it must be addressed in patients with thromboembolic illnesses. Studies should be performed that clearly differentiate the inhibitory or proliferative effect of platelet-derived molecules on tumors. For example, PF4, thrombospondin-1, HGF, Ang-1, endostatin, and PAI-1 suppress tumor growth by inhibiting angiogenesis [336]. Likewise, platelet-induced immunity also negatively affects tumor growth. Therefore, applying antiplatelet therapy for tumor treatment should be performed with caution.

### 3.6. Platelet in Coronavirus Disease 2019 (COVID-19)

COVID-19, which is caused by the severe acute respiratory syndrome coronavirus 2 (SARS-CoV-2) that wiped the globe in 2019–2020 shows serious thrombotic events, such as deep vein thrombosis, pulmonary embolism, and microthrombi, appeared as additional symptoms of the virus. Coronavirus infection causes severe thrombotic events through a variety of pathways, especially due to the result of the profound COVID-19 inflammatory response and endothelial activation rather than its procoagulant effects [337]. Initially, the spike protein of the SARS-CoV-2 virus interacts with integrin αIIbβ3 to attract platelets to the walls of endothelium and create thrombi. As a result, patients with COVID-19 have increased pathophysiological signs of inflamed endothelium in the pulmonary microcirculation (endothelitis), which causes a large increase in platelet p-selectin surface expression resulting in platelet hyperactivation and hypofibrinolysis [338,339]. Following endothelial responses, the microcirculatory arrest is worsened, resulting in organ failure and tissue damage [340]. Subsequently, the tissue damage factors such as vWF, fibrinogen, PAI1, soluble thrombomodulin, or angiopoietin caused by tissue damage are elevated in plasma and further activate platelets [341,342,343]. An increased PF4 plasma level in COVID-19 patients further expressed an elevated α- and dense granule release from platelets. Enhanced phosphorylation of the PKCδ, which is an intracellular key regulator of α- and dense granule release results in sensitization of platelet activations and secretion pathways [344].

A total of 5–41.7% of patients with COVID-19 infection also exhibit thrombocytopenia. A low plateletcrit and an elevated MPV, platelet distribution width, and platelet large cell ratio are also seen in patients with a severe COVID-19 infection [341,345,346,347]. Since the extensive manifestation of thrombotic events including microthrombi and thromboembolism in COVID-19 patients, platelets are being consumed and the megakaryocyte count and proplatelet formation in bone marrow can be increased [348]. Another reason is the SARS-CoV-2-induced-antibody mediated apoptosis of platelets in COVID-19 patients, which is mediated by immunoglobulin G (IgG) antibodies on the platelet surface via the FCr-receptor IIA (immunoreceptor tyrosine-based activation motif (ITAM)) [349]. These apoptotic processes are also influenced by elevated cytosolic calcium and increased phosphatidylserine in platelets [349,350].

The most rapid and worldwide vaccination program has been developed to combat SARS-CoV-2; however, it comes with the risk of adverse effects including immune thrombocytopenia (ITP). ITP developed by vaccination is very close to the established association between infections and autoimmunity. Thus, the International Society on Thrombosis and Hemostasis recommends antithrombotic prophylaxis using low-molecular-weight heparin, such as enoxaparin, nadroparin, dalteparin, bemiparin, and tinzaparin, for the anticoagulant, anti-inflammatory, and protecting endothelium effect [351]. Patients who have had incident thromboembolic events or have a strong suspicion of thromboembolic illness have been suggested to be benefited from therapy with a full dosage of anticoagulant [352]. Co-administration of the drug such as gamma globulin intravenous infusion (IVIg) that boosts platelet count is recommended in COVID-19 patients [353,354]. Few clinical reports have demonstrated that fibrinolytic therapy can help patients live longer by breaking down blood clots [355]. Recombinant human thrombopoietin (rhTPO), which regulates the production of platelets, can improve COVID-19-related ITP [356].

Despite these continuous discoveries and attempts, additional efforts are needed due to several variants of the SARS-CoV-2 virus. Several questions still need further investigation. For example, is thrombocytopenia induced by platelet hyperactivities and consumption during microthrombi formation in severe COVID-19 cases? Is it possible that hypercoagulable conditions interact with platelet activation, which produces phosphatidylserine and propels cell-based thrombin production, resulting in thrombosis? Do platelets in COVID-19 patients release/synthesize cytokines and contribute to the cytokine storm? Do platelets play a role in the SARS-CoV-2 immune response? Finally, during SARS-CoV-2 infection, are platelets friends or adversaries, or can they flip roles? All of these questions are vital and should be investigated further. Overall, we believe that a better understanding of platelet-pathogen interactions will elucidate the pathophysiology of infectious illnesses and that modulating platelet-pathogen interactions will open up new treatment possibilities.

## 4. Conclusions

Platelet subpopulations are a hot topic in science. Based on the diverse platelet physical, biochemical, and functional variability, the dynamic nature of platelet properties in health and disease varies. Platelet function has been studied for decades, and the same can be said for inflammation and cancer. Platelet biology overlaps with other non-thrombotic disease conditions. Nevertheless, there are significant gaps in our understanding of platelet function in these other disorders that prohibit us from reaching a thorough mechanistic understanding. Despite the fact that much of the overlap reveals antidotal links, future research is anticipated to find novel pathophysiological pathways that are extremely relevant to human disorders. We hope that this review will inspire more research into the various roles platelets play in physiological and pathological circumstances related to hemostasis and thrombosis and beyond.

## Figures and Tables

**Table 1:** Granule contents of the platelet.

Granule	Type	Contents	Role	References
**α-granules**	**Adhesive proteins**	P-selectin	Promoting adherence of leukocytes to activated platelets and endothelium	[20]
		Fibrinogen	Binding to GpIIb/IIIa receptors on the surface of platelets	[21]
		Von Willebrand factor	Binding to FVIII on the surface of platelets	[22]
		Fibronectin	Binding to integrin α5β1 and αvβ3 on the surface of platelets	[23]
		Thrombospondin-1 Thrombospondin-2	Binding to β1, αIIβ3, and αvβ3 on the surface of platelets	[24]
		Laminin-8	Binding to α3β1, and α6β1 on the surface of platelets	[25]
		Vitronectin	Binding to αvβ3 on the surface of platelets, and uPAR	[26]
	**Growth factors**	Epidermal growth factor (EGF)	Stimulating the proliferation of fibroblasts and epithelial cells	[27]
		Insulin-like growth factor 1 (IGF-1)	The major mediator of growth hormone-stimulated somatic growth and growth hormone-independent anabolic responses	[28]
		Hepatocyte growth factor (HGF)	Metabolic flux of glucose in different insulin-sensitive cell types; plays a key role in β-cell homeostasis	[29]
		Transforming growth factor β (TGF-β)	Inhibiting the proliferation of epithelial cells	[30]
		Platelet-derived growth factor (PDGF)	Growth control of mesenchymal cells such as fibroblasts and smooth muscle cells	[31]
	**Angiogenic factors**	Vascular endothelium growth factor (VEGF)	Enhanced endothelial cell proliferation and survival, increased migration and invasion of endothelial cells, increased permeability of existing vessels, forming a lattice network for endothelial cell migration, enhanced chemotaxis, and homing of bone marrow-derived vascular precursor cells	[32]
		Platelet-derived growth factor (PDGF)	Up-regulating VEGF production, modulating the proliferation and recruitment of perivascular cells	[33]
		Fibroblast growth factor (FGF)	Activating a serine-rich proteins/serine-rich phosphorylating kinases network that regulates VEGFR1 alternative spicing in endothelial cells.	[34]
	**Chemokines**	CXCL8 (IL 8) CXCL7 (platelet basic protein/NAP-2) CXCL1 (GRO- α) CXCL5 (ENA-78) CXCL2 (MIP-2) CXCL6 (LIX) CXCL-12 (SDF-1α)	Activating and recruiting neutrophils	[35] [36] [37] [38] [39] [40] [41]
		CCL5 (RANTES)	Recruiting T cells, macrophages, eosinophils, and basophils	[42]
		CCL3 (MIP-1α)	Recruiting polymorphonuclear leukocyte	[43]
		CCL2 (MCP-1)	Recruiting polymorphonuclear leukocyte	[43]
		CCL7 (MCP-3)	Recruiting monocytes, neutrophils, eosinophils, and basophils	[44]
		IL1β	Recruiting and activating leukocytes	[45]
		CD40L Proteases	-	
	**Coagulation factors**	Factor V	Cleaving prothrombin to thrombin	[46]
		Protein S	Anticoagulant by inhibiting FIXa	[47]
		Factor XI	Hemostasis through activation of factor IX	[48]
		Factor XIII	Stabilizing fibrin networks	[49]
		Kininogens	Activating FXI	[50]
		Plasminogen	Fibrinolysis by binding to the fibrin clot	[51]
	**Integral membrane proteins**	Integrin αIIbβ3	By binding fibrinogen, facilitates irreversible binding of platelets to the exposed extracellular matrix and enables the cross-linking of adjacent platelets	[52]
		GPIba-IX-V	By binding the von Willebrand factor, initiating platelet aggregation and thrombus formation	[53]
		GPVI	By binding collagen, initiating platelet aggregation	[54]
		TLT-1	Binds fibrinogen and plays a role in bleeding initiated by inflammatory insults	[55]
		P-selectin	Promoting adherence of leukocytes to activated platelets and endothelium	[20]
	**Immune mediators**	Complement C3 precursor	Cleaved into C3a and C3b by foreign invaders, and triggering inflammation, phagocytosis, cell lysis, and cell activation	[56]
		Complement C4 precursor	Activating classical and lectin pathways and the formation of C3 convertase	[56]
		Factor D	Initiating the alternative pathway of complement activation and amplification loop of C3 activation	[57]
		Factor H	Controlling the alternative pathway	[58]
		C1 inhibitor	Inhibiting the activation of the proteins of early blood coagulation and the classical complement pathways	[59]
		Immunoglobulins	Binding and neutralizing antigens	[60]
	**Protease inhibitors**	α2-antiplasmin	Inhibiting plasminogen binding to fibrin, and cross-linking fibrin	[61]
		PAI-1	Binding and inhibiting the tissue-type and urokinase-type plasminogen activator	[62]
		α2-antitrypsin	Anti-inflammatory properties by the destructive effect of major proteases	[63]
		α2-macroglobulin	Binding foreign peptides, thereby serving as humoral defense barriers against pathogens	[64]
		TFPI	Inhibiting FXa and FVIIa, thereby blocking the initial steps of the extrinsic coagulation pathway	[65]
		C1-inhibitor	Inhibiting the activation of the proteins of early blood coagulation and the classical complement pathways	[59]
	**Proteoglycans**	MMP2, MMP9	Degrading collagen, elastin, fibronectin, gelatin, and laminin and remodeling the extracellular matrix	[66]
**Dense granules**	**Amines**	Serotonin	Inducing constriction of injured blood vessels and enhancing platelet aggregation to minimize blood loss	[67]
		Histamine	Aggregatory and immunological stimuli	[68]
	**Bivalent cations**	Ca^2+^ Mg^2+^		
	**Nucleotides**	ATP ADP GTP GDP		
	**Polyphosphates**			
**Lysosome granules**	**Acid proteases**	Cathepsin D, E Carboxypeptidases (A, B) Prolinecarboxypeptidase Collagenase Acid phosphatase Arylsulphatase		
	**Glycohydrolases**	Heparinase β-*N*-acetyl-glucosaminidase		

vWF, von Willebrand factor; EGF, epidermal growth factor; IGF-1, insulin-like growth factor 1; HGF, hepatocyte growth factor; TGF-β, transforming growth factor β; PDGF, platelet-derived growth factor; VEGF, vascular endothelial growth factor; FGF, fibroblast growth factor; PF4, platelet factor 4; CXCL, C-X-C motif chemokine ligand; IL, interleukin; NAP-2, neutrophil-activating peptide 2; GRO-α, growth-related oncogene α; ENA, epithelial-neutrophil activating peptide; MIP, macrophage inflammatory protein; LIX, lipopolysaccharide-induced CXC chemokine; SDF-1α, Stromal derived factor 1α; CCL, C-C motif chemokine ligand; RANTES, regulated upon activation, normal T cell expressed and presumably secreted; MCP, Monocyte chemoattractant protein; TLT-1, TREM-like transcript 1; PAI-1, plasminogen activator inhibitor 1; TFPI, tissue factor pathway inhibitor.

**Table 2:** Platelet molecules, their functions, and pathways involved in CVD. Adapted and modified from [71].

Function	Platelet Molecule	Mechanism	References
	Thrombin	Strengthn platelet-fibrin clot	[72]
Platelet activation molecule	Nitric oxide	Enhance thromboxane generation and platelet aggregation	[73]
	LDL	Increase ROS formation Platelet activation Prothrombinase complex thrombin and thrombosis formation	[74,75,76]
	WDR1	Increase ADF-cofilin Actin disassembling Decrease actin cytoskeleton and thrombin activation	[77,78,79]
	Leptin	Increase PDE3A Decrease cAMP	[80,81]
	Adiponectin	Insulin resistance Hypoadiponectinemia	[82,83,84,85]
	ROS	8-isoPGF2α increase Platelets aggregation Drugs’ non-responsiveness	[86,87,88,89,90]
	ADP	Increase platelet activation Active leukocytes, endothelial cells, and SMCs	[91,92,93]
	Fractalkine	Increase platelet activation Inducing P-selectin Platelet adhesion to fibrinogen and collagen	[94,95]
	GPVI	Platelet aggregation Initiated signaling cascades with SFKs	[96,97]
	Integrin αIIbβ3	Platelet aggregation Initiated signaling cascades with SFKs	[96,97]
	Talin	Bind and regulate αIIbβ3	[96,97]
	ILK	Integrin activation α-granule secretion and platelet activation	[98,99,100]
	MPs	Induce angiogenesis and revascularization improvement	[101,102,103]
	SPARC	Angiogenesis formation Cardiac integrity Platelet reorganization	[101,102,103]
	miRNA-223	Regulate EPB41L3 gene ACS formation and vascular dysfunction	[104]
	miRNA-126	Regulate VCAM-1 gene ACS formation and vascular dysfunction	[104]
Molecule activation by platelet	Thromboxane	ROS generation	[73]
	Annexin V	Inducing cardiac myocytes apoptosis	[105]
	P-selectin	By linking with PSGL-1, induce several inflammation, such as myocardial infarction, stroke, and peripheral artery diseases.	[106]
	CD40L	Induce plaque initiation, thrombus stabilization, platelet activation, and vascular inflammation	[107]
	PDGF-D	Stimulates proliferation of cardiac interstitial fibroblasts and arterial smooth muscle cells.	[108]
	PDGF-A PDGF-B	Induce cardiac hypertrophy and fibrosis mediate by mesenchymal Fibroblasts	[109]
	Tissue factor	Initiating the coagulation cascade Induce migration and proliferation of vascular smooth muscle cells	[110]
	PECAM-1	Regulating NF-kB-mediated gene expression	[111]
	Peroxisome proliferator-activated receptor gamma (PPAR-r)	Induce insulin resistance, Increase vasodilation	[112]
	ACC	Fatty acid metabolism in platelets	[113,114]
	AMPK	Changes in platelet shape and secrete the granules	[113,114]
	MLCs	Changes in platelet shape and secrete the granules	[113,114]
	Cofilin	Changes in platelet shape and secrete the granules	[113,114]
	Thrombin	AMPK activation in platelets and phosphorylation of MLCs and cofilin	[113,114]
	CXCL7	Most expressions in platelet Role in CVD	[70]
	CXCL4	Increased atherosclerosis Heterodimer with CCL5 Bind to monocytes Modulate signaling pathways in the cells	[115,116,117]
	CCL5	Increase the monocytes and T-lymphocytes adhesion by ICAM-1 and VCAM-1 Increase angiogenesis	[118]
	CXCL1	Bind to CXCR2 Induce monocyte activation to atherosclerotic lesions	[119]
	CXCL12	Atherosclerotic lesions formation Activate platelets via CXCR4 Secrete by macrophages and increase the atherosclerosis	[120,121]
	CCL2	Trigger atherosclerotic lesions formation	[70,122]
	CCL3	Express in atherosclerosis Bind to CCR1 and CCR5	[70,122]

LDL, low-density lipoprotein; ROS, reactive oxygen species; WDR1, WD repeat protein 1; ADF, actin-depolymerizing factor; PDE3A, phosphodiesterase 3A; cAMP, cyclic AMP; 8-isoPGF2α, 8-iso-prostaglandin F2α; ADP, adenosine diphosphate; SMC, smooth muscle cell; ILK, integrin-linked kinases; MP, microparticle; SPARC, secreted protein acidic and rich in cysteine; miRNA, microRNA; EPB41L3, erythrocyte membrane protein band 4.1 like 3; ACS, acute coronary syndrome; VCAM-1, vascular cell adhesion molecule 1; ACC, acetyl CoA carboxylase; AMPK, AMP-activated protein kinase; MLC, myosin light chain; CXCL, C-X-C motif chemokine ligand; CVD, cardiovascular disease; CCL, C-C motif chemokine ligand; ICAM-1, intercellular adhesion molecule 1; CXCR, C-X-C motif chemokine receptor; CCR, C-C motif chemokine receptor.

**Table 3:** Platelet-derived mediators of inflammation. The table has been modified from [162].

Mediator	Main Interactions	Main Role in Inflammation
P-selectin (CD62P)	Monocytes Neutrophils Endothelium	Formation of PLA [163] Formation of bridges between leukocytes and endothelium [164]
CD40L (CD154)	T cells B cells Monocytes DCs Endothelial cells	Important mediator of T cell immune response [165] Link between innate and adaptive immune responses [165] Promotes leukocyte recruitment to the endothelium [166]
PF4 (CXCL4)	Monocytes Neutrophils	Induces leukocyte pro-inflammatory cytokine release, phagocytosis, chemotaxis, generation of ROS [122,167,168] Inhibits leukocyte apoptosis [167] Promote neutrophil firm adhesion on the endothelium [169]
MIP-1α (CCL3)	Monocytes Macrophages Neutrophil	Promotes monocyte, macrophage, and neutrophil chemotaxis [170] Upregulates monocyte and macrophage release of pro-inflammatory mediators [170]
RANTES (CCL5)	Monocytes Macrophages T cells Endothelial cells	Promotes monocyte, macrophage, and T cell chemotaxis and recruitment to the endothelium [171,172] Induce expression of MMP [173]
IL-1	SMC Monocyte Macrophage Endothelium T cells	Central to pro-inflammatory cytokine cascade and vascular inflammation [174] Increase the expression of adhesion factors on endothelial cells to enable migration [175] Vasodilation and hypotension Increase the expansion of naïve and memory CD4 T cells [176]
Microbicidal proteins	Bacteria	Disrupt cell membrane [177]
NAP-2 (CXCL7)	Neutrophil	Promote neutrophil firm adhesion on the endothelium and transmigration [169]
SDF-1α	Monocyte	Regulating leukocyte polarization and motility [178]
Serotonin	Neutrophil T-cell	Neutrophil and T-cell recruitment, vasodilation, and increasing vascular permeability [179]
Histamine	Endothelium	Vasodilation, increasing vascular permeability, and endothelial activation [180]

PLA, platelet-leukocyte aggregation; DC, dendritic cell; PF, platelet factor; CXCL, C-X-C motif chemokine ligand; ROS, reactive oxygen species; MIP, macrophage inflammatory protein; CCL, C-C motif chemokine ligand; RANTES, regulated upon activation, normal T cell expressed and presumably secreted; MMP, matrix metalloproteinase; IL, interleukin; SMC, smooth muscle cell; NAP-2, neutrophil-activating peptide 2; SDF, Stromal derived factor.

## Abbreviations

GF	Growth factor
PAMPs	Pathogen-associated molecular patterns
NO	Nitric oxide
PGI2	Prostacyclin
vWF	Von Willebrand factor
GP	Glycoprotein
ADP	Adenosine diphosphate
TxA2	Thromboxane A2
VEGF	Vascular endothelial growth factor
FGF	Fibroblast growth factor
PDGF	Platelet derived growth factor
MMP	Matrix metalloproteinase
EMT	Epithelial-mesenchymal transition
PF	Platelet factor
RANTES	Regulated upon activation, normal T cell expressed and presumably secreted
IL	Interleukin
EGF	Epidermal growth factor
HGF	Hepatocyte growth factor
TGF-β	Transforming growth factor β
CXCL	C-X-C motif chemokine ligand
SDF-1α	Stromal derived factor 1α
CCL	C-C motif chemokine ligand
MCP	Monocyte chemoattractant protein
CD	Cluster of differentiation
TLT-1	TREM-like transcript 1
Ig	Immunoglobulin
PAI-1	Plasminogen activator inhibitor 1
*TFPI*	*Tissue factor pathway inhibitor*
ATP	Adenosine triphosphate
GTP	Guanosine triphosphate
GDP	Guanosine diphosphate
CVD	Cardiovascular disease
LDL	Low-density lipoprotein
ROS	Reactive oxygen species
WDR1	WD repeat protein 1
PDE3A	Phosphodiesterase 3A
cAMP	Cyclic adenosine monophosphate
SMC	Smooth muscle cell
SFKs	Src family kinases
MPs	Microparticles
MiRNA	MicroRNA
ACS	Acute coronary syndrome
VCAM-1	Vascular cell adhesion molecule 1
ICAM-1	Intercellular adhesion molecule 1
CXCR	C-X-C Motif Chemokine Receptor
MRP	Myeloid-related protein
PAFR	Platelet-activating factor receptor
LEPRL	Leptin activates long-form leptin receptor
JAK2	Janus kinase 2
PI3K	Phosphatidylinositol 3-kinase
PKB	Protein kinase B
IRS-1	Insulin receptor substrate-1
PLA	Platelet-leukocyte aggregation
ASA	Acetylsalicylic acid
DM	Diabetes mellitus
PKC	Protein kinase C
TNF-α	Tumor necrosis factor α
T2DM	Type 2 diabetes mellitus
Mac-1	Macrophage antigen 1
PRP	Platelet Rich Plasma
VEGFR	Vascular endothelial growth factor receptor
PEDF	Pigment epithelium-derived factor
DIC	Disseminated intravascular coagulation
NETs	Neutrophil extracellular traps
TLRs	*Toll-like receptors*
β-TG	β-thromboglobulin
SrpA	Serine-rich protein A
RGD	Arginine–glycine–aspartic acid
Clf	Clumping factors
*TFPI*	*Cyclooxygenase-1*
SIRS	Systemic inflammatory response syndrome
DAMPs	Damage-related molecular patterns
PSGL-1	P-selectin glycoprotein ligand-1
PAF	Platelet activating factor
MS	Multiple sclerosis
AD	Alzheimer’s disease
mTOR	Mammalian target of rapamycin
MAPK	Mitogen-activated protein kinase
CANTOS	Canakinumab anti-inflammatory thrombosis outcome study
DC	Dendritic cell
CLEC-2	C-type lectin-like receptor II-type
MHC	Major histocompatibility complex
CNS	Central nervous system
BBB	Blood-brain barrier
EAE	Experimentally induced autoimmune encephalomyelitis
CSF	Cerebrospinal fluid
PSD95	Postsynaptic density protein95
CVT	Cerebral venous thrombosis
tMCAO	Transient middle cerebral artery occlusion
MPV	Mean platelet volume
DSCG	Disodium cromoglycate
G-CSF	Granulocyte colony-stimulating factor
GM-CSF	Granulocyte-macrophage colony-stimulating factors
RhoA	Ras homolog family member A
MYPT1	Myosin phosphatase target subunit 1
*PP1*	*Protein phosphatase 1*
YAP 1	Yes-associated protein 1
SMAD	Suppressor of Mothers Against Decapentaplegic
*NF-kB*	*Nuclear factor kappa B*
COVID-19	Coronavirus disease 2019
SARS-CoV-2	severe acute respiratory syndrome coronavirus 2
ITAM	Immunoreceptor tyrosine-based activation motif
ITP	Immune thrombocytopenia
IVIg	Gamma globulin intravenous infusion
rhTPO	Recombinant human thrombopoietin

## References

[1] Harvey J.W. (2017). The feline blood film. J. Feline Med. Surg..

[2] Hayashi T., Tanaka S., Hori Y., Hirayama F., Sato E., Inoue M. (2011). Role of mitochondria in the maintenance of platelet function during in vitro storage. Transfus. Med..

[3] Melchinger H., Jain K., Tyagi T., Hwa J. (2019). Role of platelet mitochondria: Life in a nucleus-free zone. Front. Cardiovasc. Med..

[4] Senzel L., Gnatenko D.V., Bahou W.F. (2009). The platelet proteome. Curr. Opin. Hematol..

[5] White J.G., Key N.S., King R.A., Vercellotti G.M. (2004). The White platelet syndrome: A new autosomal dominant platelet disorder. Platelets.

[6] Amable P.R., Carias R.B., Teixeira M.V., da Cruz Pacheco I., Corrêa do Amaral R.J., Granjeiro J.M., Borojevic R. (2013). Platelet-rich plasma preparation for regenerative medicine: Optimization and quantification of cytokines and growth factors. Stem Cell Res. Ther..

[7] Bambace N.M., Holmes C.E. (2011). The platelet contribution to cancer progression. J. Thromb. Haemost..

[8] Rendu F., Brohard-Bohn B. (2001). The platelet release reaction: Granules’ constituents, secretion and functions. Platelets.

[9] Thon J.N., Peters C.G., Aslam R., Rowley J., Weyrich A.S., Semple J.W., Flaumenhaft R.C., Italiano J.E. (2011). The Functional Role of TLR9 in Human Platelets. Blood.

[10] Sharda A., Flaumenhaft R. (2018). The life cycle of platelet granules. F1000Research.

[11] Gawaz M., Vogel S. (2013). Platelets in tissue repair: Control of apoptosis and interactions with regenerative cells. Blood.

[12] Jennings L.K. (2009). Mechanisms of platelet activation: Need for new strategies to protect against platelet-mediated atherothrombosis. Thromb. Haemost..

[13] Becker R.C., Sexton T., Smyth S.S. (2018). Translational implications of platelets as vascular first responders. Cir. Res..

[14] Uchimido R., Schmidt E.P., Shapiro N.I. (2019). The glycocalyx: A novel diagnostic and therapeutic target in sepsis. Crit. Care.

[15] Periayah M.H., Halim A.S., Saad A.Z.M. (2017). Mechanism action of platelets and crucial blood coagulation pathways in hemostasis. Int. J. Hematol. Oncol. Stem Cell Res..

[16] Nieswandt B., Brakebusch C., Bergmeier W., Schulte V., Bouvard D., Mokhtari-Nejad R., Lindhout T., Heemskerk J.W., Zirngibl H., Fässler R. (2001). Glycoprotein VI but not α2β1 integrin is essential for platelet interaction with collagen. EMBO J..

[17] Offermanns S. (2006). Activation of platelet function through G protein–coupled receptors. Cir. Res..

[18] Palta S., Saroa R., Palta A. (2014). Overview of the coagulation system. Indian J. Anaesth..

[19] Alberio L., Safa O., Clemetson K.J., Esmon C., Dale G. (2000). Surface expression and functional characterization of α-granule factor V in human platelets: Effects of ionophore A23187, thrombin, collagen, and convulxin. Blood.

[20] Hayashi S.-i., Watanabe N., Nakazawa K., Suzuki J., Tsushima K., Tamatani T., Sakamoto S., Isobe M. (2000). Roles of P-selectin in inflammation, neointimal formation, and vascular remodeling in balloon-injured rat carotid arteries. Circulation.

[21] Sørensen B., Tang M., Larsen O.H., Laursen P.N., Fenger-Eriksen C., Rea C.J. (2011). The role of fibrinogen: A new paradigm in the treatment of coagulopathic bleeding. Thromb. Res..

[22] Peyvandi F., Garagiola I., Baronciani L. (2011). Role of von Willebrand factor in the haemostasis. Blood Transfus..

[23] Huveneers S., Truong H., Fässler R., Sonnenberg A., Danen E.H. (2008). Binding of soluble fibronectin to integrin α5β1–link to focal adhesion redistribution and contractile shape. J. Cell Sci..

[24] Zhang K., Li M., Yin L., Fu G., Liu Z. (2020). Role of thrombospondin-1 and thrombospondin-2 in cardiovascular diseases. Int. J. Mol. Med..

[25] Fujiwara H., Kikkawa Y., Sanzen N., Sekiguchi K. (2001). Purification and Characterization of Human Laminin-8: Laminin-8 stimulates cell adhesion and migration through α3β1 AND α6β1integrins. J. Bio. Chem..

[26] Madsen C.D., Ferraris G.M.S., Andolfo A., Cunningham O., Sidenius N. (2007). uPAR-induced cell adhesion and migration: Vitronectin provides the key. J. Cell Biol..

[27] Wong R.W.C., Guillaud L. (2004). The role of epidermal growth factor and its receptors in mammalian CNS. Cytokine Growth Factor Rev..

[28] Clemmons D.R., Snyder P., Martin K. (2014). Physiology of Insulin-Like Growth Factor 1. https://www.uptodate.com/contents/physiology-of-insulin-like-growth-factor-1.

[29] Oliveira A.G., Araújo T.G., Carvalho B.d.M., Rocha G.Z., Santos A., Saad M.J. (2018). The role of hepatocyte growth factor (HGF) in insulin resistance and diabetes. Front. Endocrinol..

[30] Nagaraj N.S., Datta P.K. (2010). Targeting the transforming growth factor-β signaling pathway in human cancer. Expert Opin. Investig. Drugs.

[31] Kardas G., Daszyńska-Kardas A., Marynowski M., Brząkalska O., Kuna P., Panek M. (2020). Role of platelet-derived growth factor (PDGF) in asthma as an immunoregulatory factor mediating airway remodeling and possible pharmacological target. Front. Pharmacol..

[32] Niu G., Chen X. (2010). Vascular endothelial growth factor as an anti-angiogenic target for cancer therapy. Curr. Drug Targets.

[33] Raica M., Cimpean A.M. (2010). Platelet-derived growth factor (PDGF)/PDGF receptors (PDGFR) axis as target for antitumor and antiangiogenic therapy. Pharmaceuticals.

[34] Jia T., Jacquet T., Dalonneau F., Coudert P., Vaganay E., Exbrayat-Héritier C., Vollaire J., Josserand V., Ruggiero F., Coll J.-L. (2021). FGF-2 promotes angiogenesis through a SRSF1/SRSF3/SRPK1-dependent axis that controls VEGFR1 splicing in endothelial cells. BMC Biol..

[35] Bernhard S., Hug S., Stratmann A.E.P., Erber M., Vidoni L., Knapp C.L., Thomaß B.D., Fauler M., Nilsson B., Ekdahl K.N. (2021). Interleukin 8 elicits rapid physiological changes in neutrophils that are altered by inflammatory conditions. J. Innate Immun..

[36] Brown A.J., Sepuru K.M., Sawant K.V., Rajarathnam K. (2017). Platelet-derived chemokine CXCL7 dimer preferentially exists in the glycosaminoglycan-bound form: Implications for neutrophil–platelet crosstalk. Front. Immunol..

[37] Sawant K.V., Poluri K.M., Dutta A.K., Sepuru K.M., Troshkina A., Garofalo R.P., Rajarathnam K. (2016). Chemokine CXCL1 mediated neutrophil recruitment: Role of glycosaminoglycan interactions. Sci. Rep..

[38] Disteldorf E.M., Krebs C.F., Paust H.-J., Turner J.-E., Nouailles G., Tittel A., Meyer-Schwesinger C., Stege G., Brix S., Velden J. (2015). CXCL5 drives neutrophil recruitment in TH17-mediated GN. J. Am Soc. Nephrol..

[39] Wang G., Huang W., Wang S., Wang J., Cui W., Zhang W., Lou A., Geng S., Li X. (2021). Macrophagic Extracellular Vesicle CXCL2 Recruits and Activates the Neutrophil CXCR2/PKC/NOX4 Axis in Sepsis. J. Immunol..

[40] Jovic S., Linge H., Shikhagaie M., Olin A., Lannefors L., Erjefält J., Mörgelin M., Egesten A. (2016). The neutrophil-recruiting chemokine GCP-2/CXCL6 is expressed in cystic fibrosis airways and retains its functional properties after binding to extracellular DNA. Mucosal Immunol..

[41] Isles H.M., Herman K.D., Robertson A.L., Loynes C.A., Prince L.R., Elks P.M., Renshaw S.A. (2019). The CXCL12/CXCR4 signaling axis retains neutrophils at inflammatory sites in zebrafish. Front. Immunol..

[42] Aldinucci D., Colombatti A. (2014). The inflammatory chemokine CCL5 and cancer progression. Mediat. Inflamm..

[43] Xue M.L., Thakur A., Cole N., Lloyd A., Stapleton F., Wakefield D., Willcox M.D. (2007). A critical role for CCL2 and CCL3 chemokines in the regulation of polymorphonuclear neutrophils recruitment during corneal infection in mice. Immunol. Cell Biol..

[44] Ford J., Hughson A., Lim K., Bardina S.V., Lu W., Charo I.F., Lim J.K., Fowell D.J. (2019). CCL7 is a negative regulator of cutaneous inflammation following Leishmania major infection. Front. Immunol..

[45] Kaneko N., Kurata M., Yamamoto T., Morikawa S., Masumoto J. (2019). The role of interleukin-1 in general pathology. Inflamm. Regen..

[46] Lam W., Moosavi L. (2019). Physiology, Factor V.

[47] Pilli V., Plautz W., Majumder R. (2016). The journey of protein S from an anticoagulant to a signaling molecule. JSM Biochem. Mol. Biol..

[48] Emsley J., McEwan P.A., Gailani D. (2010). Structure and function of factor XI. Blood Am. J. Hematol..

[49] Shaz B.H., Hillyer C.D. (2013). Transfusion Medicine and Hemostasis: Clinical and Laboratory Aspects.

[50] Wu Y. (2015). Contact pathway of coagulation and inflammation. Thromb. J..

[51] Baker S.K., Strickland S. (2020). A critical role for plasminogen in inflammation. J. Exp. Med..

[52] Albert F., Christopher N.F. (2012). The platelet fibrinogen receptor: From megakaryocyte to the mortuary. JRSM Cardiovasc. Dis..

[53] Andrews R.K., Gardiner E.E., Shen Y., Whisstock J.C., Berndt M.C. (2003). Glycoprotein Ib–IX–V. Int. J. Biochem. Cell Biol..

[54] Moroi M., Jung S.M. (2004). Platelet glycoprotein VI: Its structure and function. Thromb. Res..

[55] Schmoker A.M., Pearson L.M.P., Cruz C., Flores L.G.C., Branfeild S., Torres F.D.P., Fonseca K., Cantres Y.M., Ramirez C.A.S., Melendez L.M. (2020). Defining the TLT-1 interactome from resting and activated human platelets. J. Proteom..

[56] Girardi G., Lingo J.J., Fleming S.D., Regal J.F. (2020). Essential role of complement in pregnancy: From implantation to parturition and beyond. Front. Immunol..

[57] Biesma D.H., Hannema A.J., van Velzen-Blad H., Mulder L., van Zwieten R., Kluijt I., Roos D. (2001). A family with complement factor D deficiency. J. Clin. Investig..

[58] Ferreira V.P., Pangburn M.K., Cortés C. (2010). Complement control protein factor H: The good, the bad, and the inadequate. Mol. Immunol..

[59] Schmaier A.H., Smith P.M., Colman R.W. (1985). Platelet C1-inhibitor. A secreted alpha-granule protein. J. Clin. Investig..

[60] Schroeder H.W., Cavacini L. (2010). Structure and function of immunoglobulins. J. Allergy Clin. Immunol..

[61] Maron B.A., Loscalzo J. (2007). The role of platelets in fibrinolysis. Platelets.

[62] Damare J., Brandal S., Fortenberry Y.M. (2014). Inhibition of PAI-1 antiproteolytic activity against tPA by RNA aptamers. Nucleic Acid Ther..

[63] Bergin D.A., Hurley K., McElvaney N.G., Reeves E.P. (2012). Alpha-1 antitrypsin: A potent anti-inflammatory and potential novel therapeutic agent. Arch. Immunol. Ther. Exp..

[64] Borth W. (1992). α2 Macroglobulin, a multifunctional binding protein with targeting characteristics. FASEB J..

[65] Kato H. (1996). Tissue factor pathway inhibitor; its structure, function and clinical significance. Pol. J. Pharmacol..

[66] Cancemi P., Aiello A., Accardi G., Caldarella R., Candore G., Caruso C., Ciaccio M., Cristaldi L., Di Gaudio F., Siino V. (2020). The role of matrix metalloproteinases (MMP-2 and MMP-9) in ageing and longevity: Focus on sicilian long-living individuals (LLIs). Mediat. Inflamm..

[67] Duerschmied D., Bode C. (2009). The role of serotonin in haemostasis. Hämostaseologie.

[68] Mannaioni P., Di Bello M., Raspanti S., Gambassi F., Mugnai L., Masini E. (1992). Platelet histamine: Characterization of the proaggregatory effect of histamine in human platelets. Int. Arch. Allergy. Immunol..

[69] Montenont E., Echagarruga C., Allen N., Araldi E., Suarez Y., Berger J.S. (2016). Platelet WDR1 suppresses platelet activity and is associated with cardiovascular disease. Blood.

[70] Gleissner C.A. (2012). Platelet-derived chemokines in atherogenesis: What’s new?. Curr. Vasc. Pharmacol..

[71] Khodadi E. (2020). Platelet function in cardiovascular disease: Activation of molecules and activation by molecules. Cardiovasc. Toxicol..

[72] Lv H.-C., Wu H.-Y., Yin J.-S., Ge J.-B. (2017). Thrombin induced platelet-fibrin clot strength in relation to platelet volume indices and inflammatory markers in patients with coronary artery disease. Oncotarget.

[73] Smyth E.M. (2010). Thromboxane and the thromboxane receptor in cardiovascular disease. Clin.l Lipidol..

[74] Chatterjee M., Rath D., Schlotterbeck J., Rheinlaender J., Walker-Allgaier B., Alnaggar N., Zdanyte M., Müller I., Borst O., Geisler T. (2017). Regulation of oxidized platelet lipidome: Implications for coronary artery disease. Eur. Heart J..

[75] Akkerman J.W.N. (2008). From low-density lipoprotein to platelet activation. Int. J. Biochem. Cell Biol..

[76] Salomon R.G. (2012). Structural identification and cardiovascular activities of oxidized phospholipids. Cir. Res..

[77] Stellos K., Ruf M., Sopova K., Kilias A., Rahmann A., Stamatelopoulos K., Jorbenadze R., Geisler T., Gawaz M., Bigalke B. (2011). Plasma levels of stromal cell-derived factor-1 in patients with coronary artery disease: Effect of clinical presentation and cardiovascular risk factors. Atherosclerosis.

[78] Kile B.T., Panopoulos A.D., Stirzaker R.A., Hacking D.F., Tahtamouni L.H., Willson T.A., Mielke L.A., Henley K.J., Zhang J.-G., Wicks I.P. (2007). Mutations in the cofilin partner Aip1/Wdr1 cause autoinflammatory disease and macrothrombocytopenia. Blood.

[79] Kueh H.Y., Charras G.T., Mitchison T.J., Brieher W.M. (2008). Actin disassembly by cofilin, coronin, and Aip1 occurs in bursts and is inhibited by barbed-end cappers. J. Cell Biol..

[80] Karpatkin S. (1969). Heterogeneity of human platelets: II. Functional evidence suggestive of young and old platelets. J. Clin. Investig..

[81] Elbatarny H.S., Netherton S.J., Ovens J.D., Ferguson A.V., Maurice D.H. (2007). Adiponectin, ghrelin, and leptin differentially influence human platelet and human vascular endothelial cell functions: Implication in obesity-associated cardiovascular diseases. Eur. J. Pharmacol..

[82] Shoji T., Koyama H., Fukumoto S., Maeno T., Yokoyama H., Shinohara K., Emoto M., Shoji T., Yamane T., Hino M. (2006). Platelet activation is associated with hypoadiponectinemia and carotid atherosclerosis. Atherosclerosis.

[83] Trovati M., Anfossi G. (2002). Influence of insulin and of insulin resistance on platelet and vascular smooth muscle cell function. J. Diabetes Complicat..

[84] Michelson A.D., Barnard M.R., Krueger L.A., Valeri C.R., Furman M.I. (2001). Circulating monocyte-platelet aggregates are a more sensitive marker of in vivo platelet activation than platelet surface P-selectin: Studies in baboons, human coronary intervention, and human acute myocardial infarction. Circulation.

[85] Elbatarny H.S., Maurice D.H. (2005). Leptin-mediated activation of human platelets: Involvement of a leptin receptor and phosphodiesterase 3A-containing cellular signaling complex. Am. J. Physiol. Endocrinol. Metabol..

[86] Fuentes E., Palomo I. (2016). Role of oxidative stress on platelet hyperreactivity during aging. Life Sci..

[87] Dayal S., Wilson K.M., Motto D.G., Miller F.J., Chauhan A.K., Lentz S.R. (2013). Hydrogen peroxide promotes aging-related platelet hyperactivation and thrombosis. Circulation.

[88] Pastori D., Pignatelli P., Carnevale R., Violi F. (2015). Nox-2 up-regulation and platelet activation: Novel insights. Prostagland. Other Lipid Mediat..

[89] Pereira J., Soto M., Palomo I., Ocqueteau M., Coetzee L.-M., Astudillo S., Aranda E., Mezzano D. (2002). Platelet aging in vivo is associated with activation of apoptotic pathways: Studies in a model of suppressed thrombopoiesis in dogs. Thromb. Haemost..

[90] Koyama H., Maeno T., Fukumoto S., Shoji T., Yamane T., Yokoyama H., Emoto M., Shoji T., Tahara H., Inaba M. (2003). Platelet P-selectin expression is associated with atherosclerotic wall thickness in carotid artery in humans. Circulation.

[91] Flierl U., Bauersachs J., Schäfer A. (2015). Modulation of platelet and monocyte function by the chemokine fractalkine (CX 3 CL 1) in cardiovascular disease. Eur. J. Clin. Investig..

[92] Parodi G., Marcucci R., Valenti R., Gori A.M., Migliorini A., Giusti B., Buonamici P., Gensini G.F., Abbate R., Antoniucci D. (2011). High residual platelet reactivity after clopidogrel loading and long-term cardiovascular events among patients with acute coronary syndromes undergoing PCI. JAMA.

[93] Mangiacapra F., De Bruyne B., Muller O., Trana C., Ntalianis A., Bartunek J., Heyndrickx G., Di Sciascio G., Wijns W., Barbato E. (2010). High residual platelet reactivity after clopidogrel: Extent of coronary atherosclerosis and periprocedural myocardial infarction in patients with stable angina undergoing percutaneous coronary intervention. JACC: Cardiovas. Interv..

[94] Lievens D., von Hundelshausen P. (2011). Platelets in atherosclerosis. Thromb. Haemost..

[95] Semple J.W., Italiano J.E., Freedman J. (2011). Platelets and the immune continuum. Nat. Rev. Immunol..

[96] Schulz C., Schäfer A., Stolla M., Kerstan S., Lorenz M., von Brühl M.-L., Schiemann M., Bauersachs J., Gloe T., Busch D.H. (2007). Chemokine fractalkine mediates leukocyte recruitment to inflammatory endothelial cells in flowing whole blood: A critical role for P-selectin expressed on activated platelets. Circulation.

[97] Schäfer A., Schulz C., Fraccarollo D., Tas P., Leutke M., Eigenthaler M., Seidl S., Heider P., Ertl G., Massberg S. (2007). The CX3C chemokine fractalkine induces vascular dysfunction by generation of superoxide anions. Arteroscler. Thromb. Vasc. Biol..

[98] Flierl U., Fraccarollo D., Lausenmeyer E., Rosenstock T., Schulz C., Massberg S., Bauersachs J., Schäfer A. (2012). Fractalkine activates a signal transduction pathway similar to P2Y12 and is associated with impaired clopidogrel responsiveness. Arteroscler. Thromb. Vasc. Biol..

[99] Postea O., Vasina E.M., Cauwenberghs S., Projahn D., Liehn E.A., Lievens D., Theelen W., Kramp B.K., Butoi E.D., Soehnlein O. (2012). Contribution of platelet CX3CR1 to platelet–monocyte complex formation and vascular recruitment during hyperlipidemia. Arteroscler. Thromb. Vasc. Biol..

[100] Schäfer A., Schulz C., Eigenthaler M., Fraccarollo D., Kobsar A., Gawaz M., Ertl G., Walter U., Bauersachs J. (2004). Novel role of the membrane-bound chemokine fractalkine in platelet activation and adhesion. Blood.

[101] Honda S., Shirotani-Ikejima H., Tadokoro S., Maeda Y., Kinoshita T., Tomiyama Y., Miyata T. (2009). Integrin-linked kinase associated with integrin activation. Blood.

[102] Tadokoro S., Shattil S.J., Eto K., Tai V., Liddington R.C., de Pereda J.M., Ginsberg M.H., Calderwood D.A. (2003). Talin binding to integrin ß tails: A final common step in integrin activation. Science.

[103] Senis Y., Antrobus R., Severin S., Parguina A., Rosa I., Zitzmann N., Watson S., García A. (2009). Proteomic analysis of integrin αIIbβ3 outside-in signaling reveals Src-kinase-independent phosphorylation of Dok-1 and Dok-3 leading to SHIP-1 interactions. J. Thromb. Haemost..

[104] Gurbel P.A., Fox K.A., Tantry U.S., Ten Cate H., Weitz J.I. (2019). Combination antiplatelet and oral anticoagulant therapy in patients with coronary and peripheral artery disease: Focus on the COMPASS trial. Circulation.

[105] Pétillot P., Lahorte C., Bonanno E., Signore A., Lancel S., Marchetti P., Vallet B., Slegers G., Neviere R. (2007). Annexin V detection of lipopolysaccharide-induced cardiac apoptosis. Shock.

[106] Guo L., Sun G., Wang G., Ning W., Zhao K. (2015). Soluble P-selectin promotes acute myocardial infarction onset but not severity. Mol. Med. Rep..

[107] Daub S., Lutgens E., Münzel T., Daiber A. (2020). CD40/CD40L and related signaling pathways in cardiovascular health and disease—the pros and cons for cardioprotection. Int. J. Mol. Sci..

[108] Pontén A., Bergsten Folestad E., Pietras K., Eriksson U. (2005). Platelet-derived growth factor D induces cardiac fibrosis and proliferation of vascular smooth muscle cells in heart-specific transgenic mice. Cir. Res..

[109] Kalra K., Eberhard J., Farbehi N., Chong J.J., Xaymardan M. (2021). Role of PDGF-A/B Ligands in Cardiac Repair After Myocardial Infarction. Front. Cell Dev. Biol..

[110] Steffel J., Lüscher T.F., Tanner F.C. (2006). Tissue factor in cardiovascular diseases: Molecular mechanisms and clinical implications. Circulation.

[111] Harry B.L., Sanders J.M., Feaver R.E., Lansey M., Deem T.L., Zarbock A., Bruce A.C., Pryor A.W., Gelfand B.D., Blackman B.R. (2008). Endothelial cell PECAM-1 promotes atherosclerotic lesions in areas of disturbed flow in ApoE-deficient mice. Arterioscler. Thromb. Vasc. Biol..

[112] Chandra M., Miriyala S., Panchatcharam M. (2017). PPARγ and its role in cardiovascular diseases. PPAR Res..

[113] Khodadi E., Asnafi A.A., Mohammadi-Asl J., Hosseini S.A., Malehi A.S., Saki N. (2017). Evaluation of miR-21 and miR-150 expression in immune thrombocytopenic purpura pathogenesis: A case-control study. Front. Biol..

[114] Yao R., Ma Y., Du Y., Liao M., Li H., Liang W., Yuan J., Yu X., Xiao H., Liao Y. (2011). The altered expression of inflammation-related microRNAs with microRNA-155 expression correlates with Th17 differentiation in patients with acute coronary syndrome. Cel. Mol. Immunol..

[115] Gatsiou A., Boeckel J.-N., Randriamboavonjy V., Stellos K. (2012). MicroRNAs in platelet biogenesis and function: Implications in vascular homeostasis and inflammation. Curr. Vasc. Pharmacol..

[116] Widera C., Gupta S.K., Lorenzen J.M., Bang C., Bauersachs J., Bethmann K., Kempf T., Wollert K.C., Thum T. (2011). Diagnostic and prognostic impact of six circulating microRNAs in acute coronary syndrome. J. Mol. Cel. Cardiol..

[117] Siasos G., Kollia C., Tsigkou V., Basdra E.K., Lymperi M., Oikonomou E., Kokkou E., Korompelis P., Papavassiliou A.G. (2013). MicroRNAs: Novel diagnostic and prognostic biomarkers in atherosclerosis. Curr. Top. Med. Chem..

[118] Bartel D.P. (2009). MicroRNAs: Target recognition and regulatory functions. Cell.

[119] Onselaer M.B., Oury C., Hunter R., Eeckhoudt S., Barile N., Lecut C., Morel N., Viollet B., Jacquet L.M., Bertrand L. (2014). The C a2+/calmodulin-dependent kinase kinase β-AMP-activated protein kinase-α1 pathway regulates phosphorylation of cytoskeletal targets in thrombin-stimulated human platelets. J. Thromb. Haemost..

[120] Pula G., Schuh K., Nakayama K., Nakayama K.I., Walter U., Poole A.W. (2006). PKCδ regulates collagen-induced platelet aggregation through inhibition of VASP-mediated filopodia formation. Blood.

[121] Pitsilos S., Hunt J., Mohler E.R., Prabhakar A.M., Poncz M., Dawicki J., Khalapyan T.Z., Wolfe M.L., Fairman R., Mitchell M. (2003). Platelet factor 4 localization in carotid atherosclerotic plaques: Correlation with clinical parameters. Thromb. Haemost..

[122] Von Hundelshausen P., Koenen R.R., Sack M., Mause S.F., Adriaens W., Proudfoot A.E., Hackeng T.M., Weber C. (2005). Heterophilic interactions of platelet factor 4 and RANTES promote monocyte arrest on endothelium. Blood.

[123] Dann R., Hadi T., Montenont E., Boytard L., Alebrahim D., Feinstein J., Allen N., Simon R., Barone K., Uryu K. (2018). Platelet-Derived MRP-14 Induces Monocyte Activation in Patients with Symptomatic Peripheral Artery Disease. J. Am. Col. Cardiol..

[124] Gleissner C.A., von Hundelshausen P., Ley K. (2008). Platelet chemokines in vascular disease. Arter. Thromb. Vasc. Biol..

[125] Chatterjee M., Gawaz M. (2013). Platelet-derived CXCL 12 (SDF-1α): Basic mechanisms and clinical implications. J. Thromb. Haemost..

[126] Weber C. (2005). Platelets and chemokines in atherosclerosis: Partners in crime. Circ. Res..

[127] Chen M., Kakutani M., Naruko T., Ueda M., Narumiya S., Masaki T., Sawamura T. (2001). Activation-dependent surface expression of LOX-1 in human platelets. Biochem. Biophys. Res. Commun..

[128] Podrez E.A., Byzova T.V., Febbraio M., Salomon R.G., Ma Y., Valiyaveettil M., Poliakov E., Sun M., Finton P.J., Curtis B.R. (2007). Platelet CD36 links hyperlipidemia, oxidant stress and a prothrombotic phenotype. Nat. Med..

[129] Chatterjee M., von Ungern-Sternberg S.N., Seizer P., Schlegel F., Büttcher M., Sindhu N., Müller S., Mack A., Gawaz M. (2015). Platelet-derived CXCL12 regulates monocyte function, survival, differentiation into macrophages and foam cells through differential involvement of CXCR4–CXCR7. Cell Death Dis..

[130] Mallat Z., Benamer H., Hugel B., Benessiano J., Steg P.G., Freyssinet J.M., Tedgui A. (2000). Elevated levels of shed membrane microparticles with procoagulant potential in the peripheral circulating blood of patients with acute coronary syndromes. Circulation.

[131] Chu S., Becker R., Berger P., Bhatt D., Eikelboom J., Konkle B., Mohler E., Reilly M., Berger J. (2010). Mean platelet volume as a predictor of cardiovascular risk: A systematic review and meta-analysis. J. Thromb. Haemost..

[132] Powe C.E., Levine R.J., Karumanchi S.A. (2011). Preeclampsia, a disease of the maternal endothelium: The role of antiangiogenic factors and implications for later cardiovascular disease. Circulation.

[133] Walsh S.W. (1985). Preeclampsia: An imbalance in placental prostacyclin and thromboxane production. Am. J. Obstetric. Gynecol..

[134] Mangos G.J. (2006). Cardiovascular disease following pre-eclampsia: Understanding the mechanisms. J. Hypertens..

[135] Gurbel P.A., Tantry U.S. (2010). Combination antithrombotic therapies. Circulation.

[136] Bonaca M.P., Bhatt D.L., Cohen M., Steg P.G., Storey R.F., Jensen E.C., Magnani G., Bansilal S., Fish M.P., Im K. (2015). Long-term use of ticagrelor in patients with prior myocardial infarction. N. Eng. J. Med..

[137] Swieringa F., Spronk H.M.H., Heemskerk J.W.M., van der Meijden P.E.J. (2018). Integrating platelet and coagulation activation in fibrin clot formation. Res. Pract. Thromb. Haemost..

[138] Santilli F., Simeone P., Liani R. (2019). The role of platelets in diabetes mellitus. Platelets.

[139] Santilli F., Simeone P., Liani R., Davì G. (2017). Platelets and Diabetes. Platelets Thrombotic Non-Thrombotic Disorders.

[140] Alexandru N., Popov D., Sbarcea A., Amuzescu M. (2007). Platelet free cytosolic calcium concentration during ageing of type 2 diabetic patients. Platelets.

[141] Nomura S. (2009). Dynamic role of microparticles in type 2 diabetes mellitus. Curr. Diabetes Rev..

[142] Kakouros N., Rade J.J., Kourliouros A., Resar J.R. (2011). Platelet function in patients with diabetes mellitus: From a theoretical to a practical perspective. Int. J. Endocrinol..

[143] Domingueti C.P., Dusse L.M.S.A., das Graças Carvalho M., de Sousa L.P., Gomes K.B., Fernandes A.P. (2016). Diabetes mellitus: The linkage between oxidative stress, inflammation, hypercoagulability and vascular complications. J. Diabetes Complicat..

[144] Emara E., Abdel-Sater K. (2011). Beneficial effects of calcium channel blocker “Nifedipine” on abnormalities of platelets and lipid metabolism in patients with type II diabetes mellitus. J. Diabetes Metab..

[145] Guthikonda S., Alviar C.L., Vaduganathan M., Arikan M., Tellez A., DeLao T., Granada J.F., Dong J.-F., Kleiman N.S., Lev E.I. (2008). Role of reticulated platelets and platelet size heterogeneity on platelet activity after dual antiplatelet therapy with aspirin and clopidogrel in patients with stable coronary artery disease. J. Am. Coll. Cardiol..

[146] Xin G., Wei Z., Ji C., Zheng H., Gu J., Ma L., Huang W., Morris-Natschke S.L., Yeh J.-L., Zhang R. (2016). Metformin uniquely prevents thrombosis by inhibiting platelet activation and mtDNA release. Sci. Rep..

[147] Vinik A.I., Erbas T., Park T.S., Nolan R., Pittenger G.L. (2001). Platelet dysfunction in type 2 diabetes. Diabetes Care.

[148] Wilkinson H.N., Hardman M.J. (2020). Wound healing: Cellular mechanisms and pathological outcomes. Open Biol..

[149] Rodrigues M., Kosaric N., Bonham C.A., Gurtner G.C. (2019). Wound healing: A cellular perspective. Physiol. Rev..

[150] Uchiyama R., Toyoda E., Maehara M., Wasai S., Omura H., Watanabe M., Sato M. (2021). Effect of platelet-rich plasma on M1/M2 macrophage polarization. Int. J. Mol. Sci..

[151] Ho-Tin-Noé B., Boulaftali Y., Camerer E. (2018). Platelets and vascular integrity: How platelets prevent bleeding in inflammation. Blood.

[152] Eisinger F., Patzelt J., Langer H.F. (2018). The platelet response to tissue injury. Front. Med..

[153] Wang Y., Gao H., Shi C., Erhardt P.W., Pavlovsky A., A Soloviev D., Bledzka K., Ustinov V., Zhu L., Qin J. (2017). Leukocyte integrin Mac-1 regulates thrombosis via interaction with platelet GPIbα. Nat. Commun..

[154] Werner S., Grose R. (2003). Regulation of wound healing by growth factors and cytokines. Physiol. Rev..

[155] Hardwicke J., Schmaljohann D., Boyce D., Thomas D. (2008). Epidermal growth factor therapy and wound healing—Past, present and future perspectives. Surgeon.

[156] Mijiritsky E., Assaf H.D., Peleg O., Shacham M., Cerroni L., Mangani L. (2021). Use of PRP, PRF and CGF in periodontal regeneration and facial rejuvenation—A narrative review. Biology.

[157] Arora G., Arora S. (2021). Platelet-rich plasma—Where do we stand today? A critical narrative review and analysis. Dermatol. Ther..

[158] Andia I., Maffulli N. (2013). Platelet-rich plasma for managing pain and inflammation in osteoarthritis. Nat. Rev. Rheumatol..

[159] Testa G., Giardina S.M.C., Culmone A., Vescio A., Turchetta M., Cannavò S., Pavone V. (2021). Intra-articular injections in knee osteoarthritis: A review of literature. J. Funct. Morphol. Kinesiol..

[160] Jenne C., Urrutia R., Kubes P. (2013). Platelets: Bridging hemostasis, inflammation, and immunity. Int. J. Lab. Hematol..

[161] Cognasse F., Laradi S., Berthelot P., Bourlet T., Marotte H., Mismetti P., Garraud O., Hamzeh-Cognasse H. (2019). Platelet inflammatory response to stress. Front. Immunol..

[162] Thomas M.R., Storey R.F. (2015). The role of platelets in inflammation. Thromb. Haemost..

[163] Evangelista V., Manarini S., Sideri R., Rotondo S., Martelli N., Piccoli A., Totani L., Piccardoni P., Vestweber D., De Gaetano G. (1999). Platelet/polymorphonuclear leukocyte interaction: P-selectin triggers protein-tyrosine phosphorylation–dependent CD11b/CD18 adhesion: Role of PSGL-1 as a signaling molecule. Blood.

[164] Mine S., Fujisaki T., Suematsu M., Tanaka Y. (2001). Activated platelets and endothelial cell interaction with neutrophils under flow conditions. Int. Med..

[165] Elzey B.D., Schmidt N.W., Crist S.A., Kresowik T.P., Harty J.T., Nieswandt B., Ratliff T.L. (2008). Platelet-derived CD154 enables T-cell priming and protection against Listeria monocytogenes challenge. Blood.

[166] Henn V., Slupsky J.R., Gräfe M., Anagnostopoulos I., Förster R., Müller-Berghaus G., Kroczek R.A. (1998). CD40 ligand on activated platelets triggers an inflammatory reaction of endothelial cells. Nature.

[167] Scheuerer B., Ernst M., Dürrbaum-Landmann I., Fleischer J., Grage-Griebenow E., Brandt E., Flad H.-D., Petersen F. (2000). The CXC-chemokine platelet factor 4 promotes monocyte survival and induces monocyte differentiation into macrophages. Blood.

[168] Kasper B., Brandt E., Brandau S., Petersen F. (2007). Platelet factor 4 (CXC chemokine ligand 4) differentially regulates respiratory burst, survival, and cytokine expression of human monocytes by using distinct signaling pathways. J. Immunol..

[169] Pitchford S., Pan D., Welch H.C. (2017). Platelets in neutrophil recruitment to sites of inflammation. Curr. Opin. Hematol..

[170] Maurer M., Von Stebut E. (2004). Macrophage inflammatory protein-1. Int. J. Biochem. Cell Biol..

[171] Mause S.F., von Hundelshausen P., Zernecke A., Koenen R.R., Weber C. (2005). Platelet microparticles: A transcellular delivery system for RANTES promoting monocyte recruitment on endothelium. Arterioscler. Thromb. Vasc. Biol..

[172] Danese S., De La Motte C., Reyes B.M.R., Sans M., Levine A.D., Fiocchi C. (2004). Cutting edge: T cells trigger CD40-dependent platelet activation and granular RANTES release: A novel pathway for immune response amplification. J. Immunol..

[173] Krensky A.M., Ahn Y.-T. (2007). Mechanisms of disease: Regulation of RANTES (CCL5) in renal disease. Nat. Clin. Pract. Nephrol..

[174] Lindemann S., Tolley N.D., Dixon D.A., McIntyre T.M., Prescott S.M., Zimmerman G.A., Weyrich A.S. (2001). Activated platelets mediate inflammatory signaling by regulated interleukin 1β synthesis. J. Cell Biol..

[175] Lee J.G., Heur M. (2013). Interleukin-1β enhances cell migration through AP-1 and NF-κB pathway-dependent FGF2 expression in human corneal endothelial cells. Biol. Cell.

[176] Ben-Sasson S.Z., Hu-Li J., Quiel J., Cauchetaux S., Ratner M., Shapira I., Dinarello C.A., Paul W.E. (2009). IL-1 acts directly on CD4 T cells to enhance their antigen-driven expansion and differentiation. Proc. Nat. Acad. Sci. USA.

[177] Yeaman M.R., Bayer A.S., Koo S.-P., Foss W., Sullam P.M. (1998). Platelet microbicidal proteins and neutrophil defensin disrupt the Staphylococcus aureus cytoplasmic membrane by distinct mechanisms of action. J. Clin. Investig..

[178] Ghasemzadeh M., Kaplan Z.S., Alwis I., Schoenwaelder S.M., Ashworth K.J., Westein E., Hosseini E., Salem H.H., Slattery R., McColl S.R. (2013). The CXCR1/2 ligand NAP-2 promotes directed intravascular leukocyte migration through platelet thrombi. Blood.

[179] Herr N., Bode C., Duerschmied D. (2017). The effects of serotonin in immune cells. Front. Cardiovasc. Med..

[180] Ashina K., Tsubosaka Y., Nakamura T., Omori K., Kobayashi K., Hori M., Ozaki H., Murata T. (2015). Histamine induces vascular hyperpermeability by increasing blood flow and endothelial barrier disruption in vivo. PLoS ONE.

[181] Vischer U.M., Jornot L., Wollheim C.B., Theler J.-M. (1995). Reactive oxygen intermediates induce regulated secretion of von Willebrand factor from cultured human vascular endothelial cells. Blood.

[182] Marcus A.J., Broekman M.J., Drosopoulos J.H., Olson K.E., Islam N., Pinsky D.J., Levi R. (2005). In Role of CD39 (NTPDase-1) in thromboregulation, cerebroprotection, and cardioprotection. Semin. Thromb. Hemost..

[183] Wagner D.D., Marder V.J. (1984). Biosynthesis of von Willebrand protein by human endothelial cells: Processing steps and their intracellular localization. J. Cell Biol..

[184] Sutton N.R., Baek A., Pinsky D.J., Mackay I.R., Rose N.R., Diamond B., Davidson A. (2014). Endothelial Cells and Inflammation. Encyclopedia of Medical Immunology: Autoimmune Diseases.

[185] Elalamy I., Chakroun T., Gerotziafas G., Petropoulou A., Robert F., Karroum A., Elgrably F., Samama M.-M., Hatmi M. (2008). Circulating platelet–leukocyte aggregates: A marker of microvascular injury in diabetic patients. Thromb. Res..

[186] Turgut B., Turgut N., Çelik Y., Tekgündüz E., Pamuk G.E., Demir M. (2011). Differences in platelet–leukocyte aggregates among subtypes of acute cerebral ischemia. J. Neurol. Sci..

[187] Evangelista V., Manarini S., Rotondo S., Martelli N., Polischuk R., McGregor J.L., De Gaetano G., Cerletti C. (1996). Platelet/polymorphonuclear leukocyte interaction in dynamic conditions: Evidence of adhesion cascade and cross talk between P-selectin and the beta 2 integrin CD11b/CD18. Blood.

[188] Wang H.-B., Wang J.-T., Zhang L., Geng Z.H., Xu W.-L., Xu T., Huo Y., Zhu X., Plow E.F., Chen M. (2007). P-selectin primes leukocyte integrin activation during inflammation. Nat. Immunol..

[189] Weber C., Springer T.A. (1997). Neutrophil accumulation on activated, surface-adherent platelets in flow is mediated by interaction of Mac-1 with fibrinogen bound to alphaIIbbeta3 and stimulated by platelet-activating factor. J. Clin. Investig..

[190] Simon D.I., Chen Z., Xu H., Li C.Q., Dong J.-F., McIntire L.V., Ballantyne C.M., Zhang L., Furman M.I., Berndt M.C. (2000). Platelet glycoprotein Ibα is a counterreceptor for the leukocyte integrin Mac-1 (CD11b/CD18). J. Exp. Med..

[191] Ghasemzadeh M., Hosseini E. (2013). Platelet-leukocyte crosstalk: Linking proinflammatory responses to procoagulant state. Thromb. Res..

[192] Plescia J., Altieri D.C. (1996). Activation of Mac-1 (CD11b/CD18)-bound factor X by released cathepsin G defines an alternative pathway of leucocyte initiation of coagulation. Biochem. J..

[193] Müller F., Mutch N.J., Schenk W.A., Smith S.A., Esterl L., Spronk H.M., Schmidbauer S., Gahl W.A., Morrissey J.H., Renné T. (2009). Platelet polyphosphates are proinflammatory and procoagulant mediators in vivo. Cell.

[194] Amor S., Peferoen L.A., Vogel D.Y., Breur M., van der Valk P., Baker D., van Noort J.M. (2014). Inflammation in neurodegenerative diseases—An update. Immunology.

[195] Flad H.-D., Brandt E. (2010). Platelet-derived chemokines: Pathophysiology and therapeutic aspects. Cell. Mol. Life Sci..

[196] Massberg S., Enders G., Matos F.C.d.M., Tomic L.I.D., Leiderer R., Eisenmenger S., Messmer K., Krombach F. (1999). Fibrinogen deposition at the postischemic vessel wall promotes platelet adhesion during ischemia-reperfusion in vivo. Blood.

[197] Katz J.N., Kolappa K.P., Becker R.C. (2011). Beyond thrombosis: The versatile platelet in critical illness. Chest.

[198] Pigozzi L., Aron J.P., Ball J., Cecconi M. (2016). Understanding platelet dysfunction in sepsis. Intensive Care Med..

[199] Nieswandt B., Kleinschnitz C., Stoll G. (2011). Ischaemic stroke: A thrombo-inflammatory disease?. J. Physiol..

[200] Czapiga M., Kirk A.D., Lekstrom-Himes J. (2004). Platelets deliver costimulatory signals to antigen-presenting cells: A potential bridge between injury and immune activation. Exp. Hematol..

[201] Silva-Cardoso S.C., Affandi A.J., Spel L., Cossu M., Van Roon J.A., Boes M., Radstake T.R. (2017). CXCL4 exposure potentiates TLR-driven polarization of human monocyte-derived dendritic cells and increases stimulation of T cells. J. Immunol..

[202] Klockenbusch C., Walsh G.M., Brown L.M., Hoffman M.D., Ignatchenko V., Kislinger T., Kast J. (2014). Global proteome analysis identifies active immunoproteasome subunits in human platelets. Mol. Cell. Proteom..

[203] Semple J.W., Speck E.R., Milev Y.P., Blanchette V., Freedman J. (1995). Indirect allorecognition of platelets by T helper cells during platelet transfusions correlates with anti-major histocompatibility complex antibody and cytotoxic T lymphocyte formation. Blood.

[204] Boegel S., Löwer M., Bukur T., Sorn P., Castle J.C., Sahin U. (2018). HLA and proteasome expression body map. BMC Med. Gen..

[205] Saunders R.N., Metcalfe M.S., Nicholson M.L. (2001). Rapamycin in transplantation: A review of the evidence. Kidney Int..

[206] Śledź K.M., Moore S.F., Durrant T.N., Blair T.A., Hunter R.W., Hers I. (2020). Rapamycin restrains platelet procoagulant responses via FKBP-mediated protection of mitochondrial integrity. Biochem. Pharmacol..

[207] Ridker P.M., Everett B.M., Thuren T., MacFadyen J.G., Chang W.H., Ballantyne C., Fonseca F., Nicolau J., Koenig W., Anker S.D. (2017). Antiinflammatory therapy with canakinumab for atherosclerotic disease. N. Engl. J. Med..

[208] Lu W.-J., Lin K.-C., Huang S.-Y., Thomas P.A., Wu Y.-H., Wu H.-C., Lin K.-H., Sheu J.-R. (2014). Role of a Janus kinase 2-dependent signaling pathway in platelet activation. Thromb. Res..

[209] Yellaturu C.R., Rao G.N. (2003). Cytosolic phospholipase A2 is an effector of Jak/STAT signaling and is involved in platelet-derived growth factor BB-induced growth in vascular smooth muscle cells. J. Biol. Chem..

[210] Suades R., Padró T., Alonso R., Mata P., Badimon L. (2013). Lipid-lowering therapy with statins reduces microparticle shedding from endothelium, platelets and inflammatory cells. Thromb. Haemost..

[211] El-Gamal H., Parray A.S., Mir F.A., Shuaib A., Agouni A. (2019). Circulating microparticles as biomarkers of stroke: A focus on the value of endothelial-and platelet-derived microparticles. J. Cell. Physiol..

[212] Fitzgerald D.J. (2001). Vascular biology of thrombosis: The role of platelet-vessel wall adhesion. Neurology.

[213] Coller B.S. (2002). A Brief and Highly Selective History of Ideas. Platelets.

[214] Semeraro N., Ammollo C.T., Semeraro F., Colucci M. (2012). Sepsis, thrombosis and organ dysfunction. Thromb. Res..

[215] Rittirsch D., Flierl M.A., Ward P.A. (2008). Harmful molecular mechanisms in sepsis. Nat. Rev. Immunol..

[216] Beristain-Covarrubias N., Perez-Toledo M., Flores-Langarica A., Zuidscherwoude M., Hitchcock J.R., Channell W.M., King L.D., Thomas M.R., Henderson I.R., Rayes J. (2019). Salmonella-induced thrombi in mice develop asynchronously in the spleen and liver and are not effective bacterial traps. Blood.

[217] Engelmann B., Massberg S. (2013). Thrombosis as an intravascular effector of innate immunity. Nat. Rev. Immunol..

[218] Levi M., Löwenberg E.C. (2008). In thrombocytopenia in critically ill patients. Semin. Thromb. Hemost..

[219] Shibazaki M., Nakamura M., Endo Y. (1996). Biphasic, organ-specific, and strain-specific accumulation of platelets induced in mice by a lipopolysaccharide from Escherichia coli and its possible involvement in shock. Infect. Immun..

[220] Andonegui G., Kerfoot S.M., McNagny K., Ebbert K.V., Patel K.D., Kubes P. (2005). Platelets express functional Toll-like receptor-4. Blood.

[221] Claushuis T.A., van Vught L.A., Scicluna B.P., Wiewel M.A., Klein Klouwenberg P.M., Hoogendijk A.J., Ong D.S., Cremer O.L., Horn J., Franitza M. (2016). Thrombocytopenia is associated with a dysregulated host response in critically ill sepsis patients. Blood.

[222] Tsirigotis P., Chondropoulos S., Frantzeskaki F., Stamouli M., Gkirkas K., Bartzeliotou A., Papanikolaou N., Atta M., Papassotiriou I., Dimitriadis G. (2016). Thrombocytopenia in critically ill patients with severe sepsis/septic shock: Prognostic value and association with a distinct serum cytokine profile. J. Crit. Care.

[223] Aslam R., Speck E.R., Kim M., Crow A.R., Bang K.A., Nestel F.P., Ni H., Lazarus A.H., Freedman J., Semple J.W. (2006). Platelet Toll-like receptor expression modulates lipopolysaccharide-induced thrombocytopenia and tumor necrosis factor-α production in vivo. Blood.

[224] Handtke S., Steil L., Greinacher A., Thiele T. (2018). Toward the relevance of platelet subpopulations for transfusion medicine. Front. Med..

[225] Russwurm S., Vickers J., Meier-Hellmann A., Spangenberg P., Bredle D., Reinhart K., Lösche W. (2002). Platelet and leukocyte activation correlate with the severity of septic organ dysfunction. Shock.

[226] Salat A., Bodingbauer G., Boehm D., Murabito M., Tochkow E., Sautner T., Mueller M.R., Fuegger R. (1999). Changes of platelet surface antigens in patients suffering from abdominal septic shock. Thromb Res..

[227] Yaguchi A., Lobo F.L., Vincent J.L., Pradier O. (2004). Platelet function in sepsis. J. Thromb. Haemost..

[228] Inwald D.P., Faust S.N., Lister P., Peters M.J., Levin M., Heyderman R., Klein N.J. (2006). Platelet and soluble CD40L in meningococcal sepsis. Intensive Care Med..

[229] Mavrommatis A.C., Theodoridis T., Orfanidou A., Roussos C., Christopoulou-Kokkinou V., Zakynthinos S. (2000). Coagulation system and platelets are fully activated in uncomplicated sepsis. Crit. Care Med..

[230] Washington A.V., Gibot S., Acevedo I., Gattis J., Quigley L., Feltz R., De La Mota A., Schubert R.L., Gomez-Rodriguez J., Cheng J. (2009). TREM-like transcript-1 protects against inflammation-associated hemorrhage by facilitating platelet aggregation in mice and humans. J. Clin. Investig..

[231] Esponda O., Morales J., Aguilar A., Gomez M., Washington A.V. (2010). Clinical studies support a role for trem-like transcript-1 during the progression of sepsis. Boletin Asociacion Medica Puerto Rico.

[232] Morales J., Villa K., Gattis J., Castro W., Colon K., Lubkowski J., Sanabria P., Hunter R., Washington A.V. (2010). Soluble TLT-1 modulates platelet-endothelial cell interactions and actin polymerization. Blood Coagul. Fibrinol. Int. J. Haemost. Thromb..

[233] Derive M., Bouazza Y., Sennoun N., Marchionni S., Quigley L., Washington V., Massin F., Max J.P., Ford J., Alauzet C. (2012). Soluble TREM-like transcript-1 regulates leukocyte activation and controls microbial sepsis. J. Immunol..

[234] Gawaz M., Dickfeld T., Bogner C., Fateh-Moghadam S., Neumann F.J. (1997). Platelet function in septic multiple organ dysfunction syndrome. Intensive Care Med..

[235] Gawaz M., Fateh-Moghadam S., Pilz G., Gurland H.J., Werdan K. (1995). Platelet activation and interaction with leucocytes in patients with sepsis or multiple organ failure. Eur. J. Clin. Investig..

[236] Cox D., Kerrigan S.W., Watson S.P. (2011). Platelets and the innate immune system: Mechanisms of bacterial-induced platelet activation. J. Thromb. Haemost..

[237] Koupenova M., Vitseva O., MacKay C.R., Beaulieu L.M., Benjamin E.J., Mick E., Kurt-Jones E.A., Ravid K., Freedman J.E. (2014). Platelet-TLR7 mediates host survival and platelet count during viral infection in the absence of platelet-dependent thrombosis. Blood.

[238] Thon J.N., Peters C.G., Machlus K.R., Aslam R., Rowley J., Macleod H., Devine M.T., Fuchs T.A., Weyrich A.S., Semple J.W. (2012). T granules in human platelets function in TLR9 organization and signaling. J. Cell Biol..

[239] Clark S.R., Ma A.C., Tavener S.A., McDonald B., Goodarzi Z., Kelly M.M., Patel K.D., Chakrabarti S., McAvoy E., Sinclair G.D. (2007). Platelet TLR4 activates neutrophil extracellular traps to ensnare bacteria in septic blood. Nat. Med..

[240] Klouche M., Klinger M.H., Kühnel W., Wilhelm D. (1997). Endocytosis, storage, and release of IgE by human platelets: Differences in patients with type I allergy and nonatopic subjects. J. Allergy Clin. Immunol..

[241] Yang W.H., Heithoff D.M., Aziz P.V., Haslund-Gourley B., Westman J.S., Narisawa S., Pinkerton A.B., Millán J.L., Nizet V., Mahan M.J. (2018). Accelerated Aging and Clearance of Host Anti-inflammatory Enzymes by Discrete Pathogens Fuels Sepsis. Cell Host Microb..

[242] Grewal P.K., Aziz P.V., Uchiyama S., Rubio G.R., Lardone R.D., Le D., Varki N.M., Nizet V., Marth J.D. (2013). Inducing host protection in pneumococcal sepsis by preactivation of the Ashwell-Morell receptor. Proc. Nat. Acad. Sci. USA.

[243] Plummer C., Wu H., Kerrigan S.W., Meade G., Cox D., Ian Douglas C. (2005). A serine-rich glycoprotein of Streptococcus sanguis mediates adhesion to platelets via GPIb. Br. J. Haematol..

[244] Hartleib J., Köhler N., Dickinson R.B., Chhatwal G.S., Sixma J.J., Hartford O.M., Foster T.J., Peters G., Kehrel B.E., Herrmann M. (2000). Protein A is the von Willebrand factor binding protein on Staphylococcus aureus. Blood.

[245] Brennan M.P., Loughman A., Devocelle M., Arasu S., Chubb A.J., Foster T., Cox D. (2009). Elucidating the role of Staphylococcus epidermidis serine–aspartate repeat protein G in platelet activation. J. Thromb. Haemost..

[246] Coburn J., Leong J.M., Erban J.K. (1993). Integrin alpha IIb beta 3 mediates binding of the Lyme disease agent Borrelia burgdorferi to human platelets. Proc. Nat. Acad. Sci. USA.

[247] Siboo I.R., Cheung A.L., Bayer A.S., Sullam P.M. (2001). Clumping factor A mediates binding of Staphylococcus aureus to human platelets. Infect. Immun..

[248] Arman M., Krauel K. (2015). Human platelet IgG Fc receptor Fcγ RIIA in immunity and thrombosis. J. Thromb. Haemost..

[249] Riaz A.H., Tasma B.E., Woodman M.E., Wooten R.M., Worth R.G. (2012). Human platelets efficiently kill IgG-opsonized *E. coli*. FEMS Immunol. Med. Microbiol..

[250] Montague S.J., Delierneux C., Lecut C., Layios N., Dinsdale R.J., Lee C.S.-M., Poulter N.S., Andrews R.K., Hampson P., Wearn C.M. (2018). Soluble GPVI is elevated in injured patients: Shedding is mediated by fibrin activation of GPVI. Blood Adv..

[251] Laursen M.A., Larsen J.B., Hvas A.-M. (2018). Platelet function in disseminated intravascular coagulation: A systematic review. Platelets.

[252] Woth G., Varga A., Ghosh S., Krupp M., Kiss T., Bogár L., Mühl D. (2011). Platelet aggregation in severe sepsis. J. Thromb. Thrombolysis.

[253] Dewitte A., Lepreux S., Villeneuve J., Rigothier C., Combe C., Ouattara A., Ripoche J. (2017). Blood platelets and sepsis pathophysiology: A new therapeutic prospect in critical ill patients?. Ann. Intensive Care.

[254] Lösche W., Boettel J., Kabisch B., Winning J., Claus R.A., Bauer M. (2012). Do aspirin and other antiplatelet drugs reduce the mortality in critically ill patients?. Thrombosis.

[255] Winning J., Neumann J., Kohl M., Claus R.A., Reinhart K., Bauer M., Lösche W. (2010). Antiplatelet drugs and outcome in mixed admissions to an intensive care unit. Crit. Care Med..

[256] Thomas M.R., Outteridge S.N., Ajjan R.A., Phoenix F., Sangha G.K., Faulkner R.E., Ecob R., Judge H.M., Khan H., West L.E. (2015). Platelet P2Y12 inhibitors reduce systemic inflammation and its prothrombotic effects in an experimental human model. Atertio. Thromb. Vasc. Biol..

[257] Sossdorf M., Otto G.P., Boettel J., Winning J., Lösche W. (2013). Benefit of low-dose aspirin and non-steroidal anti-inflammatory drugs in septic patients. Crit. Care.

[258] Eisen D.P., Reid D., McBryde E.S. (2012). Acetyl salicylic acid usage and mortality in critically ill patients with the systemic inflammatory response syndrome and sepsis. Crit. Care Med..

[259] Kaur C., Ling E.A. (2008). Blood brain barrier in hypoxic-ischemic conditions. Curr. Neurovasc. Res..

[260] Zlokovic B.V. (2008). The blood-brain barrier in health and chronic neurodegenerative disorders. Neuron.

[261] Langer H.F., Chavakis T. (2013). Platelets and neurovascular inflammation. Thromb. Haemost..

[262] Behari M., Shrivastava M. (2013). Role of platelets in neurodegenerative diseases: A universal pathophysiology. Int. J. Neurosci..

[263] Sotnikov I., Veremeyko T., Starossom S.C., Barteneva N., Weiner H.L., Ponomarev E.D. (2013). Platelets recognize brain-specific glycolipid structures, respond to neurovascular damage and promote neuroinflammation. PLoS ONE.

[264] Danese E., Montagnana M., Lippi G. (2014). Platelets and migraine. Thromb. Res..

[265] D’Andrea G., Cananzi A.R., Toldo M., Ferro-Milone F. (1986). Platelet activation and migraine: A study with flunarizine. Headache.

[266] Govitrapong P., Limthavon C., Srikiatkhachorn A. (1992). 5-HT2 serotonin receptor on blood platelet of migraine patients. Headache.

[267] Zeller J.A., Frahm K., Baron R., Stingele R., Deuschl G. (2004). Platelet-leukocyte interaction and platelet activation in migraine: A link to ischemic stroke?. J. Neurol. Neurosurg. Psychiatry.

[268] Wilmshurst P.T., Nightingale S., Walsh K.P., Morrison W.L. (2005). Clopidogrel reduces migraine with aura after transcatheter closure of persistent foramen ovale and atrial septal defects. Heart.

[269] Langer H.F., Choi E.Y., Zhou H., Schleicher R., Chung K.J., Tang Z., Göbel K., Bdeir K., Chatzigeorgiou A., Wong C. (2012). Platelets contribute to the pathogenesis of experimental autoimmune encephalomyelitis. Circ. Res..

[270] Kihara Y., Ishii S., Kita Y., Toda A., Shimada A., Shimizu T. (2005). Dual phase regulation of experimental allergic encephalomyelitis by platelet-activating factor. J. Exp. Med..

[271] Callea L., Arese M., Orlandini A., Bargnani C., Priori A., Bussolino F. (1999). Platelet activating factor is elevated in cerebral spinal fluid and plasma of patients with relapsing-remitting multiple sclerosis. J. Neuroimmunol..

[272] Giles J.A., Greenhalgh A.D., Denes A., Nieswandt B., Coutts G., McColl B.W., Allan S.M. (2018). Neutrophil infiltration to the brain is platelet-dependent, and is reversed by blockade of platelet GPIbα. Immunology.

[273] Gupta P., Bigley A.B., Markofski M., Laughlin M., LaVoy E.C. (2018). Autologous serum collected 1 h post-exercise enhances natural killer cell cytotoxicity. Brain Behav. Immun..

[274] Sardi F., Fassina L., Venturini L., Inguscio M., Guerriero F., Rolfo E., Ricevuti G. (2011). Alzheimer’s disease, autoimmunity and inflammation. The good, the bad and the ugly. Autoimmun. Rev..

[275] De Silva H.A., Aronson J.K., Grahame-Smith D.G., Jobst K.A., Smith A.D. (1998). Abnormal function of potassium channels in platelets of patients with Alzheimer’s disease. Lancet.

[276] Kniewallner K.M., Ehrlich D., Kiefer A., Marksteiner J., Humpel C. (2015). Platelets in the Alzheimer’s disease brain: Do they play a role in cerebral amyloid angiopathy?. Curr. Neurovasc. Res..

[277] Kleinschnitz C., Pozgajova M., Pham M., Bendszus M., Nieswandt B., Stoll G. (2007). Targeting platelets in acute experimental stroke: Impact of glycoprotein Ib, VI, and IIb/IIIa blockade on infarct size, functional outcome, and intracranial bleeding. Circulation.

[278] Kleinschnitz C., De Meyer S.F., Schwarz T., Austinat M., Vanhoorelbeke K., Nieswandt B., Deckmyn H., Stoll G. (2009). Deficiency of von Willebrand factor protects mice from ischemic stroke. Blood.

[279] Thornton P., McColl B.W., Greenhalgh A., Denes A., Allan S.M., Rothwell N.J. (2010). Platelet interleukin-1α drives cerebrovascular inflammation. Blood.

[280] Kasperska-Zajac A., Rogala B. (2007). Platelet activation during allergic inflammation. Inflammation.

[281] Katoh N. (2009). Platelets as versatile regulators of cutaneous inflammation. J. Dermatol. Sci..

[282] Tamagawa-Mineoka R., Katoh N., Ueda E., Masuda K., Kishimoto S. (2008). Elevated platelet activation in patients with atopic dermatitis and psoriasis: Increased plasma levels of beta-thromboglobulin and platelet factor 4. Allergol. Int. Off. J. Jpn. Soc. Allergol..

[283] Benton A.S., Kumar N., Lerner J., Wiles A.A., Foerster M., Teach S.J., Freishtat R.J. (2010). Airway platelet activation is associated with airway eosinophilic inflammation in asthma. J. Investig. Med. Off. Publ. Am. Fed. Clin. Res..

[284] Pitchford S.C., Yano H., Lever R., Riffo-Vasquez Y., Ciferri S., Rose M.J., Giannini S., Momi S., Spina D., O’Connor B. (2003). Platelets are essential for leukocyte recruitment in allergic inflammation. J. Allergy Clin. Immunol..

[285] Pitchford S.C., Riffo-Vasquez Y., Sousa A., Momi S., Gresele P., Spina D., Page C.P. (2004). Platelets are necessary for airway wall remodeling in a murine model of chronic allergic inflammation. Blood.

[286] Kameyoshi Y., Schröder J.M., Christophers E., Yamamoto S. (1994). Identification of the cytokine RANTES released from platelets as an eosinophil chemotactic factor. Int. Arch. Allergy Immunol..

[287] Benveniste J., Henson P.M., Cochrane C.G. (1972). Leukocyte-dependent histamine release from rabbit platelets: The role of IgE, basophils, and a platelet-activating factor. J. Exp. Med..

[288] Johansson M.W., Han S.-T., Gunderson K.A., Busse W.W., Jarjour N.N., Mosher D.F. (2012). Platelet activation, P-selectin, and eosinophil β1-integrin activation in asthma. Am. J. Respir. Crit. Care Med..

[289] Gallagher J., Bernstein I., Maccia C., Splansky G., Glueck H. (1978). Cyclic platelet dysfunction in IgE-mediated allergy. J. Allergy Clin. Immunol..

[290] Palma-Carlos A., Palma-Carlos L., Santos C.B., de Sousa C. (1991). Platelet aggregation in allergic reactions. Int. Arch. Allergy Immunol..

[291] Szczeklik A., Milner P., Birch J., Watkins J., Martin J. (1986). Prolonged bleeding time, reduced platelet aggregation, altered PAF-acether sensitivity and increased platelet mass are a trait of asthma and hay fever. Thromb. Haemost..

[292] Kowal K., Pampuch A., Kowal-Bielecka O., DuBuske L., Bodzenta-Łukaszyk A. (2006). Platelet activation in allergic asthma patients during allergen challenge with Dermatophagoides pteronyssinus. Clin. Exp. Allergy.

[293] Taytard A., Guenard H., Vuillemin L., Bouvot J., Vergeret J., Ducassou D., Piquet Y., Freour P. (1986). Platelet kinetics in stable atopic asthmatic patients. Am. Rev. Res. Dis..

[294] Taytard A., Vuillemin L. (1987). Platelet kinetics in stable asthmatic patients. Agents Actions Suppl..

[295] Ind P., Peters A., Malik F., Lavender J., Dollery C. (1985). Pulmonary platelet kinetics in asthma. Thorax.

[296] Hemmendinger S., Pauli G., Tenabene A., Pujol J.L., Bessot J.C., Eber M., Cazenave J.-P. (1989). Platelet function: Aggregation by PAF or sequestration in lung is not modified during immediate or late allergen-induced bronchospasm in man. J. Allergy Clin. Immunol..

[297] Tunon-de-Lara J., Rio P., Marthan R., Vuillemin L., Ducassou D., Taytard A. (1992). The effect of sodium cromoglycate on platelets: An in vivo and in vitro approach. J. Allergy Clin. Immunol..

[298] Shah S.A., Page C.P., Pitchford S.C. (2017). Platelet–eosinophil interactions as a potential therapeutic target in allergic inflammation and asthma. Front. Med..

[299] Page C. (1988). The involvement of platelets in non-thrombotic processes. Trend. Pharmacol. Sci..

[300] Haemmerle M., Stone R.L., Menter D.G., Afshar-Kharghan V., Sood A.K. (2018). The platelet lifeline to cancer: Challenges and opportunities. Cancer Cell.

[301] Franco A.T., Corken A., Ware J. (2015). Platelets at the interface of thrombosis, inflammation, and cancer. Blood.

[302] Pandey A.K., Singhi E.K., Arroyo J.P., Ikizler T.A., Gould E.R., Brown J., Beckman J.A., Harrison D.G., Moslehi J. (2018). Mechanisms of VEGF (vascular endothelial growth factor) inhibitor-associated hypertension and vascular disease. Hypertension.

[303] Ucuzian A.A., Gassman A.A., East A.T., Greisler H.P. (2010). Molecular mediators of angiogenesis. J. Burn Care Res..

[304] Rouwkema J., Khademhosseini A. (2016). Vascularization and angiogenesis in tissue engineering: Beyond creating static networks. Trend. Biotechnol..

[305] Teven C.M., Farina E.M., Rivas J., Reid R.R. (2014). Fibroblast growth factor (FGF) signaling in development and skeletal diseases. Genes Dis..

[306] Shen Z., Yao C., Wang Z., Yue L., Fang Z., Yao H., Lin F., Zhao H., Sun Y.-J., Bian X.-W. (2016). Vastatin, an endogenous antiangiogenesis polypeptide that is lost in hepatocellular carcinoma, effectively inhibits tumor metastasis. Mol. Ther..

[307] O’Reilly M.S., Boehm T., Shing Y., Fukai N., Vasios G., Lane W.S., Flynn E., Birkhead J.R., Olsen B.R., Folkman J. (1997). Endostatin: An endogenous inhibitor of angiogenesis and tumor growth. Cell.

[308] Mancuso M.E., Santagostino E. (2017). Platelets: Much more than bricks in a breached wall. Br. J. Haematol..

[309] Raman D., Baugher P.J., Thu Y.M., Richmond A. (2007). Role of chemokines in tumor growth. Cancer Lett..

[310] Gay L.J., Felding-Habermann B. (2011). Contribution of platelets to tumour metastasis. Nat. Rev. Cancer.

[311] Wiesner T., Bugl S., Mayer F., Hartmann J.T., Kopp H.-G. (2010). Differential changes in platelet VEGF, Tsp, CXCL12, and CXCL4 in patients with metastatic cancer. Clin. Exp. Metastasis.

[312] Suzuki A., Takahashi T., Nakamura K., Tsuyuoka R., Okuno Y., Enomoto T., Fukumoto M., Imura H. (1992). Thrombocytosis in patients with tumors producing colony-stimulating factor. Blood.

[313] Holmes C.E., Levis J.E., Schneider D.J., Bambace N.M., Sharma D., Lal I., Wood M.E., Muss H.B. (2016). Platelet phenotype changes associated with breast cancer and its treatment. Platelets.

[314] Chadha A.S., Kocak-Uzel E., Das P., Minsky B.D., Delclos M.E., Mahmood U., Guha S., Ahmad M., Varadhachary G.R., Javle M. (2015). Paraneoplastic thrombocytosis independently predicts poor prognosis in patients with locally advanced pancreatic cancer. Acta Oncol..

[315] Monreal M., Fernandez-Llamazares J., Piñol M., Julian J.F., Broggi M., Abad A. (1998). Platelet count and survival in patients with colorectal cancer–a preliminary study. Thromb. Haemost..

[316] Jefferson K., Persad R. (2001). Poor prognosis associated with thrombocytosis in patients with renal cell carcinoma. BJU Int..

[317] Buergy D., Wenz F., Groden C., Brockmann M.A. (2012). Tumor–platelet interaction in solid tumors. Int. J. Cancer.

[318] Haemmerle M., Taylor M.L., Gutschner T., Pradeep S., Cho M.S., Sheng J., Lyons Y.M., Nagaraja A.S., Dood R.L., Wen Y. (2017). Platelets reduce anoikis and promote metastasis by activating YAP1 signaling. Nat. Commun..

[319] Carr B.I., Cavallini A., D’Alessandro R., Refolo M.G., Lippolis C., Mazzocca A., Messa C. (2014). Platelet extracts induce growth, migration and invasion in human hepatocellular carcinoma in vitro. BMC Cancer.

[320] Labelle M., Begum S., Hynes R.O. (2011). Direct signaling between platelets and cancer cells induces an epithelial-mesenchymal-like transition and promotes metastasis. Cancer Cell.

[321] Clere N., Renault S., Corre I. (2020). Endothelial-to-mesenchymal transition in cancer. Front. Cell Dev. Biol..

[322] Chen H., Lan X., Liu M., Zhou B., Wang B., Chen P. (2013). Direct TGF-β1 signaling between activated platelets and pancreatic cancer cells primes cisplatin insensitivity. Cell Biol. Int..

[323] Köck K., Grube M., Jedlitschky G., Oevermann L., Siegmund W., Ritter C.A., Kroemer H.K. (2007). Expression of adenosine triphosphate-binding cassette (ABC) drug transporters in peripheral blood cells. Clin. Pharmacokin..

[324] Huijbers E.J., van Beijnum J.R., Thijssen V.L., Sabrkhany S., Nowak-Sliwinska P., Griffioen A.W. (2016). Role of the tumor stroma in resistance to anti-angiogenic therapy. Drug Resist. Updates.

[325] Van Der Zee A.G., De Bruijn H.W., Krans M., De Cuyper E.M., Hollema H., Limburg P.C., Bijzet J., De Vries E.G. (1995). Higher levels of interleukin-6 in cystic fluids from patients with malignant versus benign ovarian tumors correlate with decreased hemoglobin levels and increased platelet counts. Cancer.

[326] Feng S., Kroll M.H., Nick A.M., Sood A.K., Afshar-Kharghan V. (2016). Platelets are not hyperreactive in patients with ovarian cancer. Platelets.

[327] Guo Y., Cui W., Pei Y., Xu D. (2019). Platelets promote invasion and induce epithelial to mesenchymal transition in ovarian cancer cells by TGF-β signaling pathway. Gynecol. Oncol..

[328] Andrade S.S., Sumikawa J.T., Castro E.D., Batista F.P., Paredes-Gamero E., Oliveira L.C., Guerra I.M., Peres G.B., Cavalheiro R.P., Juliano L. (2017). Interface between breast cancer cells and the tumor microenvironment using platelet-rich plasma to promote tumor angiogenesis-influence of platelets and fibrin bundles on the behavior of breast tumor cells. Oncotarget.

[329] Ibele G.M., Kay N.E., Johnson G.J., Jacob H.S. (1985). Human platelets exert cytotoxic effects on tumor cells. Blood.

[330] Pacchiarini L., Serra L., Grignani G., Gamba G., Gorini M. (1982). In vitro effect of culture fluids from neoplastic tissues on platelet aggregation. I. Human tumors of the gastrointestinal tract. Boll. Della Soc. Ital. Biol. Sper..

[331] Gasic G.J., Gasic T.B., Galanti N., Johnson T., Murphy S. (1973). Platelet—tumor-cell interactions in mice. The role of platelets in the spread of malignant disease. Int. J. Cancer.

[332] Mezouar S., Darbousset R., Dignat-George F., Panicot-Dubois L., Dubois C. (2015). Inhibition of platelet activation prevents the P-selectin and integrin-dependent accumulation of cancer cell microparticles and reduces tumor growth and metastasis in vivo. Int. J. Cancer.

[333] Pucci F., Rickelt S., Newton A.P., Garris C., Nunes E., Evavold C., Pfirschke C., Engblom C., Mino-Kenudson M., Hynes R.O. (2016). PF4 promotes platelet production and lung cancer growth. Cell Rep..

[334] Hicks B.M., Murray L.J., Hughes C., Cardwell C.R. (2015). Clopidogrel use and cancer-specific mortality: A population-based cohort study of colorectal, breast and prostate cancer patients. Pharmacoepidemiol. Drug Saf..

[335] Gebremeskel S., LeVatte T., Liwski R.S., Johnston B., Bezuhly M. (2015). The reversible P2Y12 inhibitor ticagrelor inhibits metastasis and improves survival in mouse models of cancer. Int. J. Cancer.

[336] Sierko E., Wojtukiewicz M.Z. (2007). Inhibition of Platelet Function: Does It Offer a Chance of Better Cancer Progression Control?. Semin. Thromb. Hemost..

[337] Connors J.M., Levy J.H. (2020). COVID-19 and its implications for thrombosis and anticoagulation. Blood.

[338] Bonaventura A., Vecchié A., Dagna L., Martinod K., Dixon D.L., Van Tassell B.W., Dentali F., Montecucco F., Massberg S., Levi M. (2021). Endothelial dysfunction and immunothrombosis as key pathogenic mechanisms in COVID-19. Nat. Rev. Immunol..

[339] Gawaz M., Langer H., May A.E. (2005). Platelets in inflammation and atherogenesis. J Clin. Investig..

[340] Nicolai L., Leunig A., Brambs S., Kaiser R., Weinberger T., Weigand M., Muenchhoff M., Hellmuth J.C., Ledderose S., Schulz H. (2020). Immunothrombotic dysregulation in COVID-19 pneumonia is associated with respiratory failure and coagulopathy. Circulation.

[341] Bi X., Su Z., Yan H., Du J., Wang J., Chen L., Peng M., Chen S., Shen B., Li J. (2020). Prediction of severe illness due to COVID-19 based on an analysis of initial Fibrinogen to Albumin Ratio and Platelet count. Platelets.

[342] Grobler C., Maphumulo S.C., Grobbelaar L.M., Bredenkamp J.C., Laubscher G.J., Lourens P.J., Steenkamp J., Kell D.B., Pretorius E. (2020). COVID-19: The Rollercoaster of Fibrin(Ogen), D-Dimer, Von Willebrand Factor, P-Selectin and Their Interactions with Endothelial Cells, Platelets and Erythrocytes. Int. J. Mol. Sci..

[343] Ruberto F., Chistolini A., Curreli M., Frati G., Marullo A.G., Biondi-Zoccai G., Mancone M., Sciarretta S., Miraldi F., Alessandri F. (2021). Von Willebrand factor with increased binding capacity is associated with reduced platelet aggregation but enhanced agglutination in COVID-19 patients: Another COVID-19 paradox?. J. Thromb. Thrombolysis.

[344] Zaid Y., Puhm F., Allaeys I., Naya A., Oudghiri M., Khalki L., Limami Y., Zaid N., Sadki K., Ben El Haj R. (2020). Platelets can associate with SARS-Cov-2 RNA and are hyperactivated in COVID-19. Circ. Res..

[345] Zhang S., Liu Y., Wang X., Yang L., Li H., Wang Y., Liu M., Zhao X., Xie Y., Yang Y. (2020). SARS-CoV-2 binds platelet ACE2 to enhance thrombosis in COVID-19. J. Hematol. Oncol..

[346] Bikdeli B., Madhavan M.V., Jimenez D., Chuich T., Dreyfus I., Driggin E., Nigoghossian C., Ageno W., Madjid M., Guo Y. (2020). Global COVID-19 Thrombosis Collaborative Group, Endorsed by the ISTH, NATF, ESVM, and the IUA, Supported by the ESC Working Group on Pulmonary Circulation and Right Ventricular Function. COVID-19 and thrombotic or thromboembolic disease: Implications for prevention, antithrombotic therapy, and follow-up: JACC state-of-the-art review. J. Am. Coll. Cardiol..

[347] Alnor A., Sandberg M.B., Toftanes B.E., Vinholt P.J. (2021). Platelet parameters and leukocyte morphology is altered in COVID-19 patients compared to non-COVID-19 patients with similar symptomatology. Scand. J. Clin. Lab. Investig..

[348] Boeckh-Behrens T., Golkowski D., Ikenberg B., Schlegel J., Protzer U., Schulz C., Novotny J., Kreiser K., Zimmer C., Hemmer B. (2021). COVID-19-associated Large Vessel Stroke in a 28-year-old Patient: NETs and Platelets Possible Key Players in Acute Thrombus Formation. Clin. Neuroradiol..

[349] Althaus K., Marini I., Zlamal J., Pelzl L., Singh A., Haberle H., Mehrlander M., Hammer S., Schulze H., Bitzer M. (2021). Antibody-induced procoagulant platelets in severe COVID-19 infection. Blood.

[350] Saleh J., Peyssonnaux C., Singh K.K., Edeas M. (2020). Mitochondria and microbiota dysfunction in COVID-19 pathogenesis. Mitochondrion.

[351] Poterucha T.J., Libby P., Goldhaber S.Z. (2017). More than an anticoagulant: Do heparins have direct anti-inflammatory effects?. Thromb. Haemost..

[352] Tang N., Bai H., Chen X., Gong J., Li D., Sun Z. (2020). Anticoagulant treatment is associated with decreased mortality in severe coronavirus disease 2019 patients with coagulopathy. J. Thromb. Haemost..

[353] Kistangari G., McCrae K.R. (2013). Immune thrombocytopenia. Hematol. Oncol. Clin..

[354] Bhattacharjee S., Banerjee M. (2020). Immune Thrombocytopenia Secondary to COVID-19: A Systematic Review. SN Compr. Clin. Med..

[355] Wang J., Hajizadeh N., Moore E.E., McIntyre R.C., Moore P.K., Veress L.A., Yaffe M.B., Moore H.B., Barrett C.D. (2020). Tissue plasminogen activator (tPA) treatment for COVID-19 associated acute respiratory distress syndrome (ARDS): A case series. J. Thromb. Haemost..

[356] Zhang X., Li M., Chen T., Lv D., Xia P., Qian W. (2021). Management of COVID-19-related immune thrombocytopenia by rhTPO. Blood Res..

